# SARS-CoV-2 infection drives an inflammatory response in human adipose tissue through infection of adipocytes and macrophages

**DOI:** 10.1126/scitranslmed.abm9151

**Published:** 2022-09-22

**Authors:** Giovanny J. Martínez-Colón, Kalani Ratnasiri, Heping Chen, Sizun Jiang, Elizabeth Zanley, Arjun Rustagi, Renu Verma, Han Chen, Jason R. Andrews, Kirsten D. Mertz, Alexandar Tzankov, Dan Azagury, Jack Boyd, Garry P. Nolan, Christian M. Schürch, Matthias S. Matter, Catherine A. Blish, Tracey L. McLaughlin

**Affiliations:** ^1^ Department of Medicine, Stanford University School of Medicine, Stanford, CA, 94305, USA; ^2^ Program in Immunology, Stanford University School of Medicine, Stanford, CA, 94305, USA; ^3^ Department of Surgery, Stanford University School of Medicine, Stanford, CA, 94305, USA; ^4^ Department of Cardiothoracic Surgery, Stanford University School of Medicine, Stanford, CA, 94305, USA; ^5^ Department of Pathology, Stanford University School of Medicine, Stanford, CA, 94305, USA; ^6^ Chan Zuckerberg Biohub, San Francisco, CA, 94158, USA; ^7^ Department of Pathology and Neuropathology, University Hospital and Comprehensive Cancer Center Tübingen, 72070, Tübingen, Germany; ^8^ Institute of Medical Genetics and Pathology, University Hospital of Basel, University of Basel, 4056, Basel, Switzerland; ^9^ Center for Virology and Vaccine Research, Beth Israel Deaconess Medical Center, Boston, MA, 02215, USA; ^10^ Institute of Pathology, Cantonal Hospital Baselland, 4410, Liestal, Switzerland

## Abstract

Obesity, characterized by chronic low-grade inflammation of the adipose tissue, is associated with adverse coronavirus disease 2019 (COVID-19) outcomes, yet the underlying mechanism is unknown. To explore whether severe acute respiratory syndrome coronavirus 2 (SARS-CoV-2) infection of adipose tissue contributes to pathogenesis, we evaluated COVID-19 autopsy cases and deeply profiled the response of adipose tissue to SARS-CoV-2 infection in vitro. In COVID-19 autopsy cases, we identified SARS-CoV-2 RNA in adipocytes with an associated inflammatory infiltrate. We identified two distinct cellular targets of infection: adipocytes and a subset of inflammatory adipose tissue-resident macrophages. Mature adipocytes were permissive to SARS-CoV-2 infection; although macrophages were abortively infected, SARS-CoV-2 initiated inflammatory responses within both the infected macrophages and bystander preadipocytes. These data suggest that SARS-CoV-2 infection of adipose tissue could contribute to COVID-19 severity through replication of virus within adipocytes and through induction of local and systemic inflammation driven by infection of adipose tissue-resident macrophages.

## Teaser

SARS-CoV-2 is capable of infecting human adipose tissue and drives inflammation.

## INTRODUCTION

Severe acute respiratory syndrome coronavirus 2 (SARS-CoV-2) has driven a global pandemic, with over 550 million cases and 6 million deaths globally as of July 2022. SARS-CoV-2 is the causative pathogenic agent of coronavirus disease-2019 (COVID-19), marked by clinical manifestations that range from asymptomatic infection to severe disease characterized by extreme inflammation and cytokine storm (*1*). Obesity, also a global pandemic largely driven by socioeconomic inequality (*2*), has emerged as an independent risk factor for infection, severe disease, and mortality (*3*–*7*). Though obesity is associated with comorbid conditions also related to severe COVID-19, the independent relative risk of obesity is higher than that of hypertension and type 2 diabetes (*3*, *6*, *8*). Further, although increased age is also a correlate of COVID-19 severity, obesity is a risk factor even in young adults and children who do not have other comorbid conditions (*9*, *10*). Obesity could contribute to poor COVID-19 outcomes through several mechanisms including impaired respiratory mechanics (*11*), an underlying metabolic milieu characterized by systemic inflammation and hypercoagulability (*12*–*14*), impaired immune responses at baseline and in response to viruses (*8*, *15*, *16*), increased risk of endotoxemia in the setting of obesity and aging (*17*–*20*), or by direct infection of adipose tissue by SARS-CoV-2 (*21*, *22*).

This latter possibility, that SARS-CoV-2 could directly infect adipose tissue, was originally hypothesized based on expression of RNA for angiotensin-converting enzyme-2 (ACE2), the SARS-CoV-2 entry receptor, in adipose tissue samples (*20*–*23*). Supporting this idea, several groups recently reported detection of SARS-CoV-2 RNA in adipose tissue from 28 of 59 COVID-19 autopsy cases, infection of human adipocytes in vitro, and infection of adipose tissue in the hamster model of SARS-CoV-2 infection (*24*–*26*). SARS-CoV-2 infection of adipose tissue could induce inflammation and alter adipose tissue function, potentially contributing to disease pathogenesis. For instance, virus infection can impact adipogenesis and could increase adipose tissue hypertrophy and hyperplasia (*27*), in turn serving as a fuel for further infection. One recent study demonstrates that COVID-19 is associated with diminished adiponectin expression in vivo, suggesting alterations of adipose tissue function (*25*). This same study also shows that in vivo infection of hamsters and in vitro infection of murine adipocytes leads to decreased adiponectin expression in adipocytes, yet it is unclear if infection is the direct cause of this change in humans because SARS-CoV-2 infection of human adipocytes in vitro did not change adiponectin expression (*25*). Adipocytes can also play a role in inflammation, secreting chemokines such as monocyte chemoattractant protein-1 (MCP-1), which can fuel macrophage infiltration and activation (*28*–*30*). Virus infection of peri-organ fat could even contribute to organ damage through inflammation and downstream processes such as extracellular matrix deposition and fibrosis, impaired cellular function, and hypercoagulability (*13*, *16*). Many questions remain unanswered, including which adipose tissue cells are infected, whether they can serve as a reservoir for continued virus production, and how infection changes inflammatory and functional profile of adipose tissue cells.

Here, we sought to answer these questions, identifying the targets of SARS-CoV-2 within adipose tissue and evaluating how infection drives inflammation and functional alterations at the single cell level. We demonstrated that SARS-CoV-2 RNA can be detected in various adipose depots from autopsy specimens from patients with COVID-19 and was associated with an inflammatory infiltrate in vivo. We found that SARS-CoV-2 abortively infects a subset of inflammatory adipose tissue-resident macrophages and productively infects adipocytes, leading to induction of inflammatory pathways even in uninfected bystander cells. Neither infected cell type consistently expresses ACE2 protein, suggesting that viral entry is mediated by an alternate entry mechanism, an important consideration for therapies aimed at ameliorating the harmful effects of SARS-CoV-2 infection of adipose tissue.

## RESULTS

### SARS-CoV-2 is detected in the adipose tissue from deceased COVID-19 patients.

**Fig. 1.**
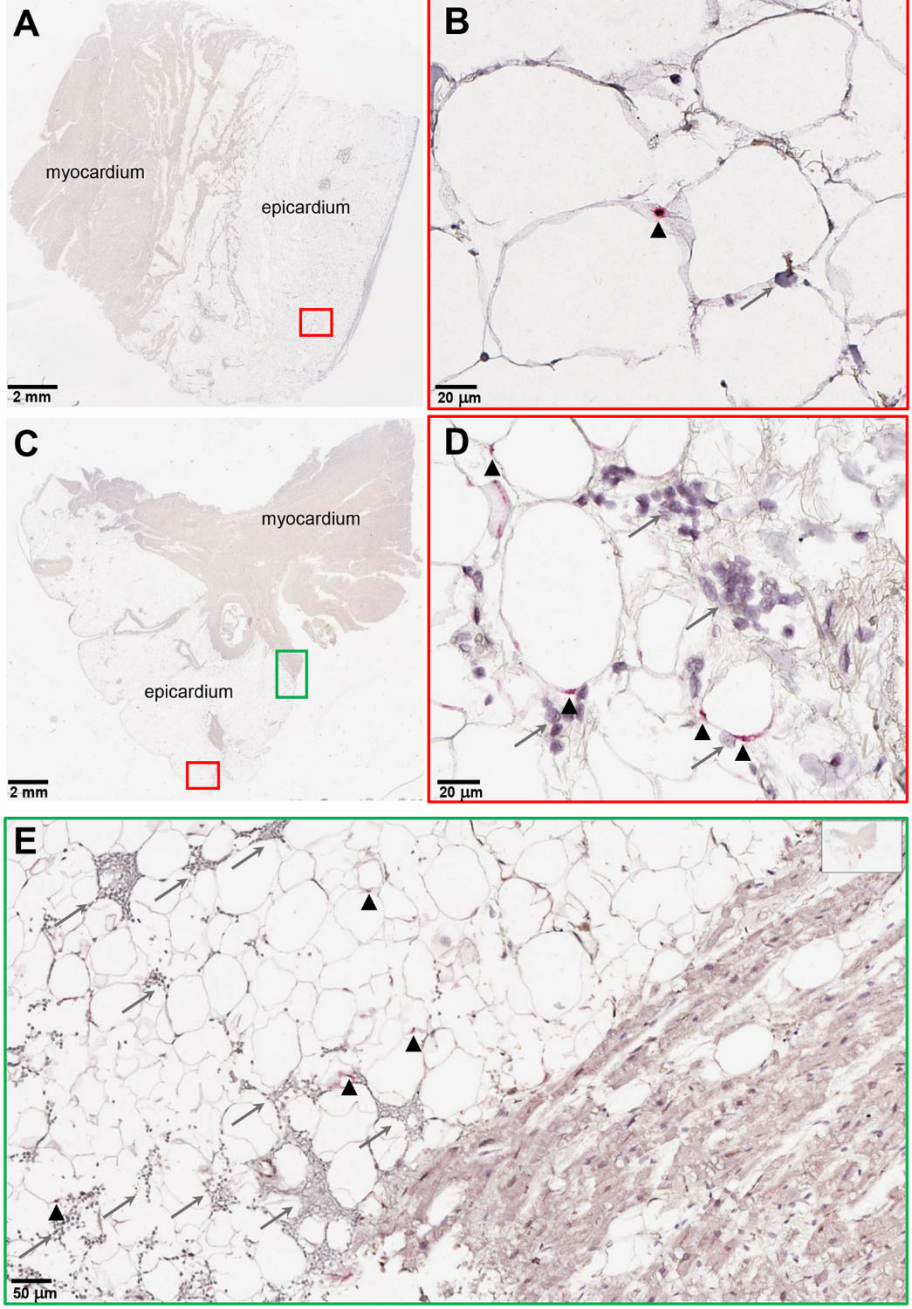
SARS-CoV-2 RNA and immune infiltration are present in adipose tissue of autopsy samples from patients with COVID-19. (**A to E**) RNA in situ hybridization (ISH) on epicardial fat from heart autopsies from patients with COVID-19 are shown. (A and B) show samples from autopsy #9. (C to E) show samples from autopsy #10. Assays were performed using probes against SARS-CoV-2 spike mRNA. Black arrowheads show ISH positive signals and gray arrows show inflammatory cells. (A and C) Overview of the heart tissue section (2mm), and (B and D) magnified view (20μm) of the represented region. (E) Interface of epicardial fat and myocardium (50μm). Note the inflammatory infiltration (gray arrows) only in the epicardial fat. Image has been rotated 90°.

To evaluate SARS-CoV-2 infection of adipose tissue in vivo, we performed real time quantitative-PCR (RTqPCR) for SARS-CoV-2 genes in adipose tissue, lung, heart, and kidney samples in a cohort of eight COVID-19 autopsy cases (Table 1; autopsy # 1 to 8). We detected SARS-CoV-2 RNA in epicardial (EAT), visceral (VAT), and subcutaneous (SAT) adipose tissue in seven of the eight patients with a mean Ct value of 33; the only patient in which we did not detect viral RNA in adipose tissue was also undetectable in lung, heart and kidney (Table 1; autopsy #1). SARS-CoV-2 detection was highest in the lung (mean Ct=25), but its expression was similar across the adipose tissue (mean Ct=33), heart (mean Ct=34) and kidney (mean Ct=34). We then performed RNA in situ hybridization (ISH) on epicardial fat and lung tissue from autopsy samples (Table 1; autopsy # 9 and 10) of patients with COVID-19 and in control SARS-CoV-2 negative individuals (Fig. 1, fig. S1). We detected SARS-CoV-2 in the cytoplasm of adipocytes in the epicardial fat with an associated mononuclear inflammatory infiltrate (Fig. 1). Thus, adipose tissue can harbor SARS-CoV-2 RNA in vivo and attract immune cells to the site of infection.

**Table 1.** SARS-CoV-2 detection in adipose tissue from deceased individuals. Quantification of SARS-CoV-2 RNA detection by PCR from autopsy cases 1 to 8. All individuals were positive for SARS-CoV-2 by nasal swab when admitted to the hospital. Threshold cycles (Ct) presented are an average of three SARS-CoV-2 gene sequences for open reading frame 1ab (*ORF1ab*), spike (*S*), and nucleocapsid (*N*). RNA was isolated from either epicardial, visceral, or subcutaneous fat. Heart RNA samples were collected from a mixture of myocardium and epicardium. Some participants were used in previously published studies assessing SARS-CoV-2 infection in lungs and heart (*31*–*33*). N/A, not available; UD, undetectable. Autopsies 9 and 10 were used for ISH as shown in Fig. 1. All Ct values presented are an average of *ORF1ab*, *S*, and *N* genes. All heart RNA samples were collected from a mixture of myocardium and epicardium. Any Ct value below 37 was considered a true SARS-CoV-2 signal. Autopsies labeled with (*) were from a separate cohort.

							RTqPCR for SARS-CoV-2 (Ct values)				
Autopsy #	Sex	Age (years)	Ethnicity	BMI (kg/m2)	DHBD (Days)	Comorbidities	Lung	Heart	Kidney	Adipose	Type of adipose analyzed
1	F	84	C	24	12	CAD, HTN, metastatic lung CA, T2D, AKI, dementia	UD	UD	UD	UD	Epicardial
2	M	85	C	26	5	HTN, dyslipidemia, T2D, prostate CA in remission, OSA, cirrhosis, leukemia in remission	23	33	33	33	Visceral
3	F	81	C	26	4	CAD, HTN, PAD, AKI, former smoker	22	34	36	36	Visceral
4	M	71	C	24	NH	PAD, CAD, valvular heart disease, AKI	27	33	UD	33	Visceral
5	M	65	C	26	8	Dyslipidemia, CAD, COPD, HTN, T2D, OSA, AKI	23	34	34	33	Epicardial
6	M	89	C	N/A	5	CAD, cardiomyopathy, dyslipidemia, T2D, OSA, former smoker, COPD	21	31	N/A	28	Epicardial
7	M	85	N/A	30	5	Former smoker, HTN, cardiomyopathy, COPD, AKI, dementia	26	35	UD	34	Subcutaneous
8	M	58	C	47	7	Former smoker, HTN, obesity, CAD, CHF, AKI	35	37	33	36	Subcutaneous
9*	F	78	C	35.2	NH	obesity, AV-block III with pacemaker, HTN, cerebellar infarcts	N/A	N/A	N/A	34	Epicardial
10*	F	87	C	N/A	1	Dementia, obesity, CAD, HTN, T2D	N/A	N/A	N/A	32	Epicardial

**Abbreviations:** days of hospitalization before death (DHBD), not hospitalized (NH), not available (N/A), Caucasian (C), male (M), female (F), body mass index (BMI), coronary artery disease (CAD), hypertension (HTN), type 2 diabetes mellitus (T2D), cancer (CA), acute renal insufficiency (AKI), obstructive sleep apnea (OSA), peripheral artery disease (PAD), chronic obstructive pulmonary disease (COPD), congestive heart failure (CHF).											

### Adipocytes are permissive to SARS-CoV-2 infection.

**Fig. 2.**
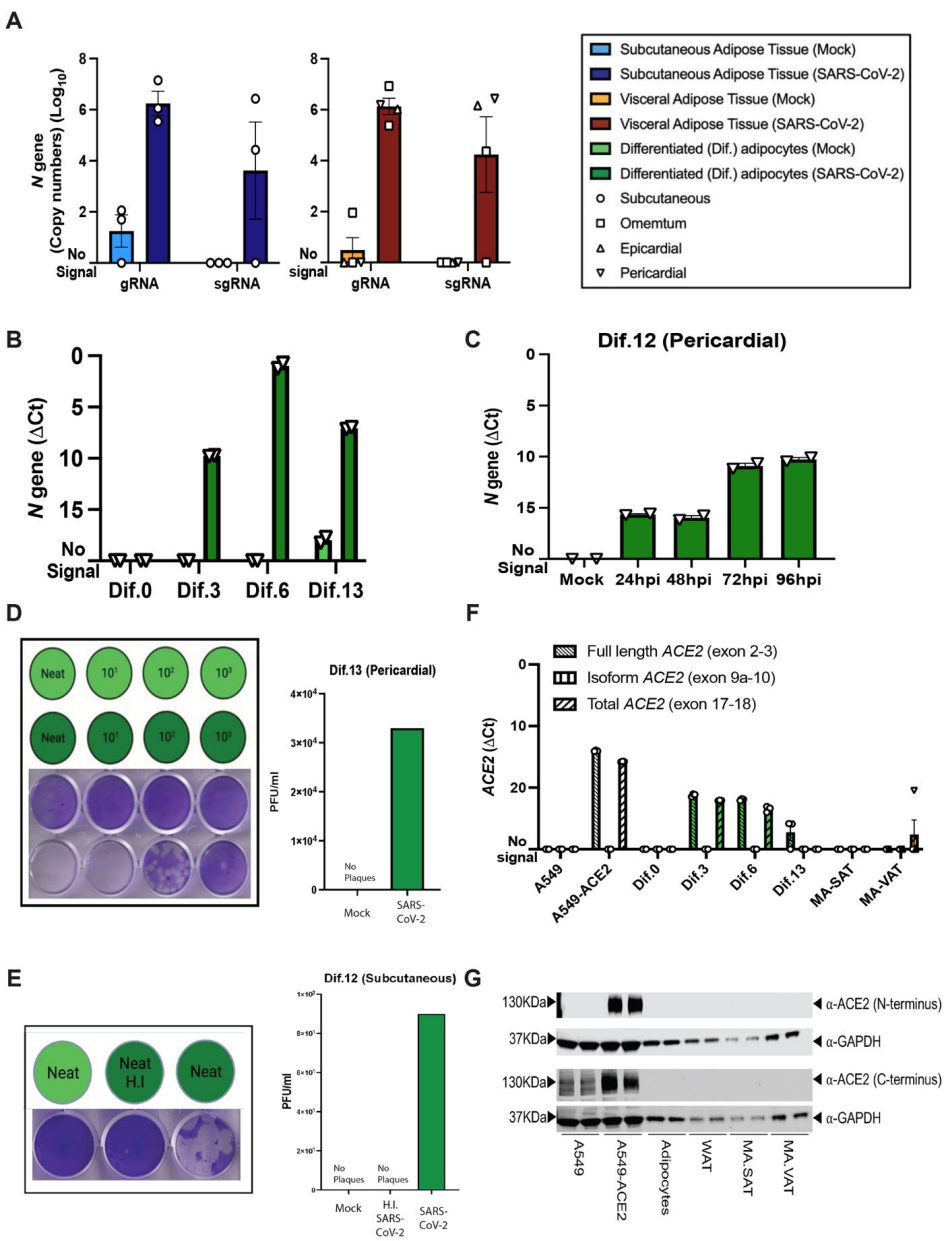
Mature and in vitro differentiated adipocytes support SARS-CoV-2 infection in the absence of ACE2 protein. Mature adipocytes (MA) from fresh human adipose tissue were isolated by collagenase digestion and infected with SARS-CoV-2 (USA-WA1/2020) at a MOI of 1, or mock-infected, for 1 hour followed by washing and culturing prior RNA collection. (**A**) Measurements of absolute SARS-CoV-2 (*N* gene) genome copy numbers of genomic (gRNA) and subgenomic (sgRNA) RNA are shown for mature adipocytes from subcutaneous (left; n=3) and visceral (right; n=2 omentum; n=1 epicardial; n=1 pericardial) adipose tissue at 24 hours post-infection (hpi). (**B**) SARS-CoV-2 *N* gene ΔCt values are shown at 24 hpi for adipocytes differentiated from pericardial adipose tissue for 0, 3, 6, and 13 days (Dif.0, 3, 6, 13). (**C**) SARS-CoV-2 *N* gene ΔCt values are shown at 24, 48, 72, and 96hpi for adipocytes differentiated from pericardial adipose tissue. In (B and C), each data point is an average of 2 technical replicates and are presented as mean ± s.e.m. (**D**) Plaque assay results are shown for supernatants collected from day 13 differentiated (Dif.13) adipocytes from pericardial adipose tissue that were either mock infected (top row, light green) or infected with SARS-CoV-2 (bottom row, dark green). Tested samples were either neat (undiluted) or serial 10-fold dilutions. Bar plot quantification of measured plaque forming units (PFU)/ml on the right. (**E**) Plaque assay results are shown for supernatant collected from day 12 differentiated (Dif.12) adipocytes from subcutaneous adipose tissue that were treated with heat inactivated (H.I) SARS-CoV-2 (middle, dark green), SARS-CoV-2 (right, dark green) or mock-infected (left, light green). Bar plot quantification of measured PFU/ml on the right. (**F**) *ACE2* RNA expression was measured in A549 cells, A549-ACE2 cells, differentiated adipocytes, whole adipose tissue (WAT), and mature adipocytes from subcutaneous (MA-SAT) and visceral (MA-VAT) adipose tissue using primers targeting the exon 2 to 3 (Full length), exon 9a to 10 (isoform), and exon 17 to 18 (total) of *ACE2.* Each data point in A549 cells, A549-ACE2 cells, differentiated adipocytes, and whole adipose tissue are averages of technical replicates. Values in MA-SAT (n=3) and MA-VAT (n=4) are from independent donors. (**G**) ACE2 protein expression was evaluated by Western blot analysis on cell types as in (F), using antibodies against the N and C terminus of ACE2. GAPDH was used as a loading control. Absolute gene quantification in (A to C) was obtained using a standard curve generated with *N* gene insert. In all cases RTqPCR analyses, ΔCt values were obtained by 1-step RTqPCR using *18s* as a housekeeping gene and data are presented as mean ± s.e.m. Color legend on the top right applies to all panels.

We recruited participants undergoing bariatric or cardiothoracic surgery (table S1), and isolated mature adipocytes from freshly harvested subcutaneous, visceral, epicardial, and pericardial (PAT) adipose tissue by collagenase digestion. Mature adipocytes were infected with SARS-CoV-2 at a multiplicity of infection (MOI) of 1 and RNA was isolated 24 hours post-infection (hpi). We detected both genomic and subgenomic SARS-CoV-2 (*N* gene*)* in infected mature adipocytes from SAT (n=3) and VAT (n=2 omentum; n=1 EAT; n=1 PAT) (Fig. 2A), providing evidence of viral RNA replication.

We next infected adipocytes differentiated from preadipocytes derived from PAT at days 0, 3, 6, and 13 of differentiation (*34*). We detected viral RNA at 24 hpi in all differentiated adipocytes, but not in the preadipocytes at day 0 (Dif.0) (Fig. 2B). In a second sample derived from SAT, we detected subgenomic and genomic RNA both in preadipocytes at day 0 and in adipocytes differentiated at day 3 (fig. S2), indicating variation in the ability of preadipocytes to be infected but consistent infection of differentiated adipocytes. Viral reads increased in differentiated adipocytes between 24 and 72 hpi, suggesting viral RNA amplification (Fig. 2C). Finally, we confirmed productive infection based on detection of plaques from the supernatant of infected preadipocyte cultures (Fig. 2D and E, fig. S3). This complements recent data demonstrating productive infection of adipocytes differentiated from mesenchymal stem cells (*26*), and indicates that adipocytes are permissive to SARS-CoV-2.

We next examined ACE2 expression, focusing both on the functional ACE2 that mediates spike protein-dependent SARS-CoV-2 cellular entry, and recently described isoforms that can be induced by inflammation but lack the receptor binding domain required for SARS-CoV-2 spike protein binding (*35*–*37*). First, we measured RNA expression using primers to detect full-length, truncated, and total isoforms, using a human adenocarcinoma cell line that does not highly express *ACE2* (A549) and one that has been genetically modified to express *ACE2* (A549-ACE2) as controls (Fig. 2F). We detected full-length *ACE2* in differentiated adipocytes, as has been previously described (*26*), but not in preadipocytes and in only one of seven mature adipocyte samples (Fig. 2F). We next assessed ACE2 protein expression, using antibodies specific for both the N- and C terminus. We did not detect ACE2 protein in any sample, whether from differentiated adipocytes, freshly isolated adipocytes, or whole fixed adipose tissue (Fig. 2G, fig. S4). Collectively, these results show that freshly isolated mature adipocytes and adipocytes differentiated in culture can be infected by SARS-CoV-2, but virus entry is likely independent of the ACE2 receptor.

### Adipose tissue macrophages are abortively infected by SARS-CoV-2.

**Fig. 3.**
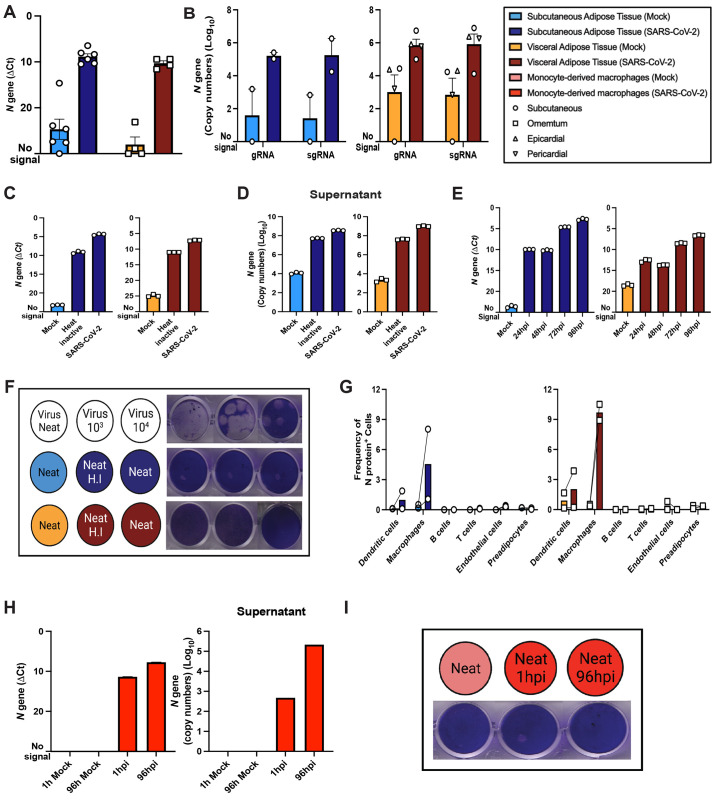
Exposure of stromal vascular cells from adipose tissue to SARS-CoV-2 leads to abortive infection in adipose tissue macrophages. Stromal vascular cells (SVC) from fresh human adipose tissue was infected with SARS-CoV-2 (USA-WA1/2020) at an MOI of 1 or mock-infected, and SARS-CoV-2 *N* gene expression was obtained by 1-step RTqPCR. (**A**) *N* gene ΔCt at 24hpi or mock (subcutaneous, n=6; visceral, n=4), and (**B**) absolute gene copy numbers of genomic (gRNA) and subgenomic (sgRNA) viral RNA at 24hpi (subcutaneous, n=2; visceral, n=4) are shown. (**C and D**) *N* gene detection on SVC exposed to SARS-CoV-2, heat inactivated SARS-CoV-2, or mock for 96hpi are reported as *N* gene ΔCt for cells (C) or N-gene absolute copy numbers for supernatant (D). (**E**) *N* gene ΔCt was measured in infected or mock SVC that were maintained for 24, 48, 72, and 96hpi before RNA isolation. (**F**) Plaque assay results are shown for neat (undiluted) and diluted viral stock (top) or neat (undiluted) supernatants collected from subcutaneous (middle, blue) or visceral (bottom, red) adipose tissue SVC from mock (left, light colors), heat inactivated SARS-CoV-2 (middle, dark colors), or SARS-CoV-2 (left, dark colors) conditions. (**G**) Frequency of SARS-CoV-2 infected cells is shown based on SARS-CoV-2 N protein detection by flow cytometry of subcutaneous (n=2) and visceral (n=2) adipose tissue. Paired samples are connected by mock (left) and infected (right). (**H**) *N* gene measurements are shown for cells (left) and supernatant (right) from monocyte-derived macrophages (MDMs) infected with SARS-CoV-2 for 1hpi, 96hpi, or mock. (**I**) Plaque assay results are shown for neat supernatant from mock infected (left, light red), and SARS-CoV-2 infected MDMs for 1 hour (middle, dark red), and 96 hours (right, dark red). Data are presented as mean ± s.e.m., except in (D) and (H, right), where data are presented in geometric means. The color legend on the top right applies to all subfigures.

To investigate whether other cells within the adipose tissue were permissive to SARS-CoV-2 infection, we infected freshly isolated stromal vascular cells (SVC) from human adipose tissue with SARS-CoV-2 at an MOI of 1. Infection was assessed by RTqPCR for SARS-CoV-2 *N-*gene, flow cytometry for N protein, and plaque assay (Fig. 3A to G). Both genomic and subgenomic viral RNA were detected in SARS-CoV-2 infected SVC from subcutaneous (n=6 Fig. 3A; n=2 Fig. 3B), omental (n=4, Fig. 3A; n=2, Fig. 3B), pericardial (n=1, Fig. 3B), and epicardial (n=1, Fig. 3B) adipose tissue. Intracellular and released viral RNA were lower when a heat-inactivated virus was used as a control (Fig. 3C and D), and viral reads accumulated over the course of 96 hours (Fig. 3E), both indicative of viral RNA replication and release. Despite viral RNA accumulation, we did not detect infectious virus in the supernatant from infected SVC from either subcutaneous or visceral tissue either by plaque assay (Fig. 3F, fig. S5). These data suggest that infection of SVC is abortive.

To identify the infected cells, we assessed viral protein production in six major SVC types (dendritic cells, macrophages, B cells, T cells, preadipocytes, and endothelial cells) by flow cytometry (Fig. 3G, fig. S6). In both subcutaneous and visceral SVC, SARS-CoV-2 N protein was primarily restricted to macrophages (Fig. 3G). The low frequency of infected macrophages (1 to 10%) raises the possibility that we may have failed to detect infectious virus release following SVC infection because there were too few infected cells. To obtain a more plentiful source of macrophages, we infected human monocyte-derived macrophages (MDMs). We detected amplification of viral RNA in infected MDMs (Fig. 3H), release of viral RNA into the supernatant (Fig. 3H), but no infectious particles in the supernatant (Fig. 3I, fig. S5), just as we observed with SVC. Collectively, these findings indicate that adipose tissue macrophages are infected and initiate viral RNA amplification and protein production, but that the infection is abortive without the production of infectious particles.

### SARS-CoV-2 infection of SVC induces inflammation in vitro.

As even abortive infection can drive inflammatory responses (*38*, *39*), we assessed the production of inflammatory mediators in infected SVC and in differentiated adipocytes. First we assessed *IL6* in SVC because of the association between interleukin (IL)-6 and low-grade inflammation in obese individuals (*40*), and the critical role of IL-6 in COVID-19 immunopathogenesis (*41*, *42*). We found that *IL6* was up-regulated in infected SVC derived from both subcutaneous and visceral tissue (fig. S7). We also assessed expression of five interferon-related genes (*IFNA1, IFNB1, ISG15, IFI27*, and *IER3*) in SVC derived from subcutaneous and visceral tissue, differentiated adipocytes, and A549-ACE2 cells at multiple time points following SARS-CoV-2 infection (fig. S8). In infected SVC, expression of *IFNA1*, *IFNB1*, and *ISG15* increased over time (fig. S8). Though *IFI27* was induced following infection of A549-ACE2 cells, it was not induced in infected SVC, nor was *IER3* induced in any sample. We observed modest induction of *IFNB1* following infection of differentiated preadipocytes, but did not observe induction of *IFNA1, ISG15, IFI27,* or *IER3.* Thus, SARS-CoV-2 infection of adipose tissue induces antiviral responses that change over time, and these responses are muted in adipocytes compared to SVC.

We also assessed production of inflammatory cytokines and chemokines in the supernatants from infected versus mock-infected cultures. As compared to mock infection, SARS-CoV-2 infection of SVC derived from subcutaneous or visceral tissue resulted in up-regulation of several inflammatory chemokines, cytokines, and growth factors (Table 2; data file S1). In both SAT and VAT, the elevated production of interferon gamma-induced protein(IP)-10 is particularly noteworthy as this cytokine is elevated in the serum of critically ill COVID-19 patients (*43*). Additionally, we found the platelet-derived growth factor (PDGF)-AA, and AB/BB (PDGFAB/BB) together with the type 2 (Th2) immune factor interleukin-4 (IL-4) to be elevated in SARS-CoV-2 infected subcutaneous SVC. Similarly, PDGF-AA, IL-4, and IL-13 (another Th2 cytokine) were elevated in the SARS-CoV-2 infected visceral SVC. We also measured infection-induced inflammatory mediators in mature adipocytes freshly isolated from subcutaneous and visceral tissue (data file S1). There were no differences in cytokine or chemokine production (data file S1). These data indicate that infection of adipose tissue by SARS-CoV-2 drives secretion of cytokines associated with severe COVID-19 infection, and that this inflammatory response is primarily mediated by the SVC.

**Table 2:** SARS-CoV-2 infection of SVC drives release of inflammatory mediators. Table summarizing results of 80-plex Luminex assay performed in supernatants of SVC (SAT, n=8; VAT, n=6) cultures that were infected with SARS-CoV-2 or mock-infected for 24 hours; cytokines that were significantly different (p < 0.05) in an unadjusted analysis are shown. First column shows analytes of interest. Second column categorizes analytes in either chemokine, growth factor, Th2 cytokine, inflammatory cytokine, cell adhesion molecule, or hematopoiesis regulatory cytokine. Columns 3 to 6 summarizes statistical results organized by mean fold change and p and q (Adjusted p value) value obtained from Wilcoxon signed rank test. Columns 7 and 8 summarizes the known role of each analyte in COVID-19. The bottom of the table contains abbreviations.

		Results					
		SAT (n=8)		VAT (n=6)			
Analyte	Category	Mean Fold change	p value/ q value	Mean Fold change	p value/ q value	Known role in COVID-19	References
IP-10	Chemokine	19.3	0.01/0.06	17.04	0.03/0.18	Elevated in acute cases and critically ill patients; associated with disease severity and ICU admission; predictor of disease progression; biomarker of impaired T cell response in acute infection	(*43*–*51*)
MCP2		0.74	0.02/0.1	1.12	0.03/0.18	Elevated in critically ill patients	(*50*)
MCP3		1.32	0.04/0.2	0.97	0.84/1	Elevated in serum and blood; associated with disease severity and predictor of disease progression	(*44*, *47*–*50*)
CXCL13		1.19	0.02/0.1	0.96	0.63/0.98	Elevated in serum	(*52*)
CCL27		0.99	0.74/0.97	1.12	0.03/0.18	Secreted by HeLa cells in response to SARS-CoV-2 ORF7a exposure	(*53*)
PDGFAA	Growth Factor	5.41	0.01/0.06	4.06	0.03/0.18	Associated with severe disease and ICU admission	(*45*)
PDGFAB/BB		1.12	0.01/0.06	1.11	0.09/0.36	Elevated in serum and associated with ICU admission	(*45*, *49*)
VEGF		3.81	0.01/0.06	2.52	0.03/0.18	Elevated in serum; role in COVID-19 brain inflammation	(*49*, *54*)
MCSF		2.57	0.01/0.06	2.07	0.03/0.18	Elevated in serum	(*46*)
GMCSF		1.81	0.08/0.31	1.49	0.03/0.18	Elevated in serum, including in mechanically ventilated patients	(*46*–*49*)
LIF		0.57	0.01/0.06	1.44	0.03/0.18	Proposed to treat COVID-19 patients by decreasing IL-6	(*55*)
TGFα		2.01	0.01/0.06	1.81	0.03/0.18	No known role	
IL-4	Th2 Cytokines	1.96	0.01/0.06	2.02	0.03/0.18	Elevated in serum	(*45*, *49*)
IL-13		1.13	0.08/0.31	1.22	0.03/0.18	Elevated in serum	(*45*)
MIF	Inflammatory cytokines	12.16	0.01/0.06	10.81	0.03/0.18	Elevated in critically ill patients	(*56*)
IL-21		1.08	0.01/0.06	0.92	0.25/0.69	Elevated in serum compared to non-COVID-19 pneumonia patients	(*57*)
TNFβ		1.17	0.01/0.06	1.23	0.03/0.18	No known role	
sICAM1	Cell adhesion	0.87	0.04/0.2	0.94	1/1	Elevated in non-survivors compared to survivors	(*58*)
SCF	Hematopoiesis regulatory cytokine	0.9	0.02/0.13	1.08	0.03/0.18	No known role	
**Abbreviations:** Transforming growth factor alpha (TGFα), Granulocyte colony stimulation factor (GMCSF), Leukemia inhibitory factor (LIF), Stem cell factor (SCF), C-C motif chemokine ligand (CCL), Soluble intercellular adhesion molecule-1 (sICAM1).							


### SARS-CoV-2 infects a distinct subset of inflammatory macrophages.

**Fig. 4.**
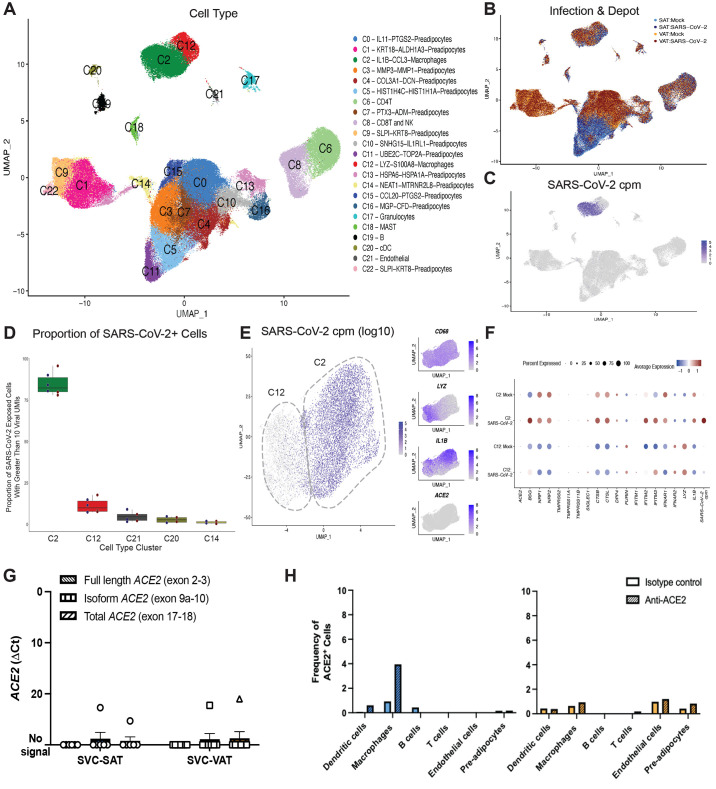
A subset of adipose tissue macrophages is infected with SARS-CoV-2. (**A to F**) SVC were isolated from the SAT and VAT depots of three different participants and were mock infected or infected with SARS-CoV-2 at an MOI of 1. Each sample was collected for scRNA-seq at 24 hpi. (**A to C**) UMAP representation of the SVC from all participants (n=3) across 198,759 cells, colored by manually annotated cell type (A), infection and depot (B), and (C) SARS-CoV-2 cpm (log10) (C). (**D**) The box and whisker plot (box defines the inter-quartile range (IQR) between first (Q1) and third (Q3) quartiles with median quartile annotated by a line, whiskers define a distance of 1.5 * IQR from Q1 and Q3) reveals the proportion of clusters within the SARS-CoV-2 infected conditions that have greater than 10 viral unique molecular identifiers (UMIs) present, showing only the top four cell clusters with the highest composition of SARS-CoV-2+ cells. (**E**) UMAP projections of all macrophages from the scRNA seq dataset are shown, colored by log10 SARS-CoV-2 cpm (left) and colored by *CD68*, *LYZ*, *IL1B* and *ACE2* expression (right). (**F**) Dotplot of the proportion of cells (dot size) in the macrophage clusters, split by infection condition, expressing genes relevant for SARS-CoV-2 entry and antiviral defense, as well as SARS-CoV-2 cpm and macrophage cluster markers, colored by scaled average expression. (**G**) *ACE2* expression was measured on SVC from SAT and VAT using primers targeting exon 2 to 3 (Full length), exon 9a to 10 (isoform), and exon 17 to 18 (total) of *ACE2*. Values are shown for SVC-SAT (n=6) and SVC-VAT (n=7). (**H**) Barplots demonstrate the frequency of ACE2-expressing cells across various cell types in the SAT (left) and the VAT (right) using flow cytometry and quantifying ACE2 expression by comparing anti-ACE2 stained cells to isotype control.

To comprehensively characterize the adipose tissue cells infected with SARS-CoV-2 and their inflammatory response, we performed single-cell RNA sequencing (scRNA-seq) of SARS-CoV-2 (24hpi) and mock-infected SVC isolated from both subcutaneous and visceral adipose tissue from three bariatric surgery patients (table S1). We generated 198,759 single-cell expression profiles. After performing dimensionality reduction and Harmony batch integration (*59*), unbiased graph-based clustering resulted in 23 cell types (C0 to C22) with marker genes that aligned with previous datasets and included three major cell groups: preadipocytes, immune cells, and endothelial cells (*60*, *61*) (Fig. 4A, data file S2, fig. S9). We saw the greatest heterogeneity across the preadipocyte population between SAT versus VAT (Fig. 4B) for which we identified 14 distinct clusters, each of which was labeled by its top two cluster defining genes. We similarly identified and labeled two distinct macrophage clusters.

We detected SARS-CoV-2 transcripts at up to 1,079 transcripts per cell and in a total of 8.8% of cells in the infected samples (fig. S10) and did not detect SARS-CoV-2 transcript in the mock-infected samples. The uniform manifold approximation and projection (UMAP) of SARS-CoV-2-exposed samples colored by viral counts-per-million (cpm) showed that macrophages contained the highest concentration of SARS-CoV-2 transcripts (Fig. 4C), with 78 to 96% of SARS-CoV-2 exposed C2-macrophages and 7 to 18% of C12-macrophages containing more than ten viral transcripts, across all participants and depots (Fig. 4D). *ACE2* transcript was not detected in the SVC macrophages and therefore could not explain the difference in SARS-CoV-2 transcripts between C2-macrophages and C12-macrophages (Fig. 4E and F). However, C2-macrophages expressed higher expression of *BSG*, *NRP1/2*, *CTSB* and *CTSL* compared to C12-macrophages (Fig. 4F), all previously noted to be important to SARS-CoV-2 infection and viral processing (*62*–*64*). To further support the lack of evidence for *ACE2* expression across the macrophages and SVC, we performed RTqPCR on both the SVC-SAT and the SVC-VAT to detect full length *ACE2* (exon 2 to 3), isoform *ACE2* (exon 9a to10), and total *ACE2* (exon 17 to 18) (Fig. 4G). Results show that *ACE2* expression was limited in SVC, with a majority of samples showing no detectable *ACE2* expression (Fig. 4G). ACE2 protein expression by flow cytometry was also limited in SVC, with no detectable expression in the SVC from visceral tissue and low expression on about 3% of macrophages derived from subcutaneous tissue when compared to isotype control (Fig. 4H, fig. S11). Additionally, we took six publicly available scRNA-seq datasets of human adipose tissue and were unable to detect notable *ACE2* expression from all 75 samples analyzed, which includes 23 samples of SAT and VAT adipocytes measured by single-nucleus sequencing (sNuc-seq) (fig. S12) (*60*, *61*, *65*). Collectively, these data suggest that entry into macrophages is likely ACE2-independent.

**Fig. 5.**
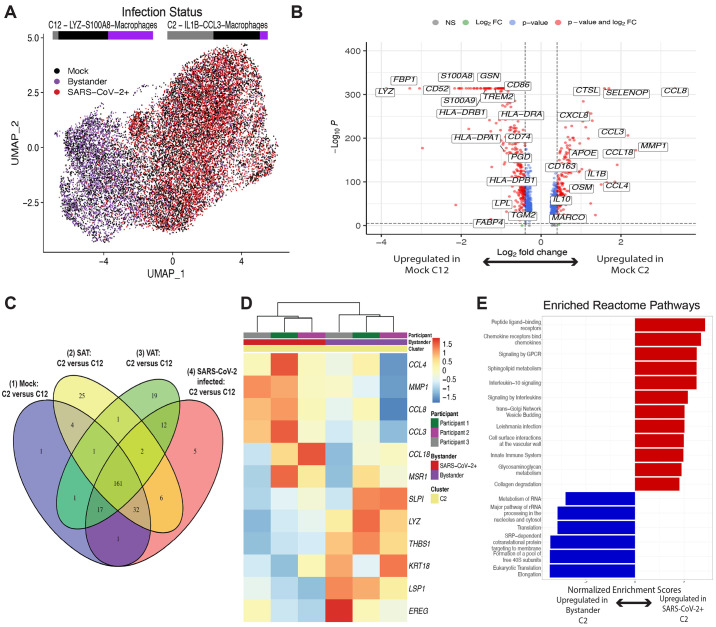
The infected macrophage cluster is marked by increased chemokine expression. (**A**) UMAP projections of a downsampled number of macrophages from the total scRNA seq dataset, such that each participant, infectious status, and depot contributed 1290 cells (the lowest number of cells across the group. Cells are colored by infection status, either Mock (black), Bystander (purple), or SARS-CoV-2+ (red). Proportion bars above each cluster are shown, representing the distribution of Infection Status across the cluster. (**B**) The volcano plot shows DEGs between macrophage clusters 2 (C2) and 12 (C12) across mock-infected samples. (**C**) A Venn diagram is shown comparing the significant DEGs (padj <.05, abs(log2FC) >= 0.6) and their direction of change across C2 versus C12 in (*1*) mock-infected, (*2*) all SAT, (*3*) all VAT, and (*4*) SARS-CoV-2-infected conditions. (**D**) The heatmap shows the most significant DEGs (padj <.001, abs(log2FC) >= 0.8) between SARS-CoV-2+ versus bystander macrophages within C2. (**E**) Reactome pathway analysis is shown for DEGs between SARS-CoV-2+ versus bystander C2 macrophages. Top ranked pathways by normalized enrichment score and padj < 0.05 are represented.

We then further evaluated how the two macrophage populations differed. We observed an even representation of mock versus infected macrophages in both clusters, indicating that infection itself did not change the composition of these clusters (Fig. 5A). We then compared the clusters between mock-infected samples to identify baseline characteristics that might contribute to the enhanced susceptibility of macrophages in the C2 cluster to infection. We identified 1000 significant differentially expressed genes (DEGs) (padj < 0.05), with up-regulation of inflammation related transcripts within C2 compared to C12, and in particular, transcripts for numerous chemokine ligands (Fig. 5B, fig. S13, A and B, data files S3 to 5). Increased expression of C-C motif chemokine ligand (CCL) *CCL8* and *CCL3*, chemokines involved in monocyte chemotaxis, highlight the potential of these infected cells to induce the infiltration of inflammatory monocytes and macrophages (*66*, *67*). There was significant (padj < 0.001) down-regulation of 14 human leukocyte antigen (HLA) genes, including *HLA-DRB1*, *HLA-DRA*, *HLA-DQB1*, and *HLA-DPB1*, in the C2-macrophages compared to C12-macrophages (Fig. 5B, data file S3). Additionally, the C2-macrophage cluster demonstrated enrichment for transcripts associated with perivascular macrophages (PVM) including *CD163*, *SELENOP*, *MARCO* and *APOE* (Fig. 5B and (*61*, *68*, *69*)). In contrast, the relatively uninfected C12 demonstrated up-regulation of genes relating to lipid metabolism and other markers of lipid-associated macrophages, including *FABP4*, *TGM2*, *GSN, TREM2, LPL* and *PGD,* suggesting that this macrophage population might play a role in lipid accumulation and trafficking (*60*, *61*, *68*, *70*). Additionally, top DEGs defining C2- versus C12- macrophages were consistent across VAT and SAT macrophages (sharing over 70% of DEGs), and both SARS-CoV-2-infected and mock-infected samples displayed a similar array of differentially expressed genes between these clusters (sharing over 85% of DEGs) (Fig. 5C).

We next determined the cell-intrinsic effects of infection, comparing infected C2-macrophages (those with SARS-CoV-2 transcripts) to uninfected bystander cells (Fig. 5D, fig. S13C, data file S6). Though there were baseline differences in gene expression between participants, several genes strongly distinguished SARS-CoV-2^+^ C2 cells from bystander C2 cells by hierarchical clustering (Fig. 5D); many of these genes also distinguish C2-macrophages from C12-macrophages (fig. S13D). For instance, *CCL4, CCL8,* and *CCL3* genes were highly enriched (padj < 0.001, log2 fold change (FC): 1.85 to 3.35) in the infected cells within C2, and *LYZ* and *THBS1* were significantly down-regulated (padj < 0.001, log2FC: -3.29, -3.87 respectively) within the infected cells. As these genes contributed to defining the C2 population from C12 cells, this result suggests that SARS-CoV-2 infection may drive C2 inflammatory features to further extremes. Pathway analysis of DEGs (padj < 0.1) between the SARS-CoV-2^+^ cells versus bystanders highlights the enrichment of pathways associated with IL-10 signaling (Fig. 5E, data file S7), which is consistent with up-regulation of this pathway in monocytes of patients with severe COVID-19 (*71*). Chemokine and other innate immune system-associated pathways are also highly enriched in infected cells (Fig. 5E), which has been previously demonstrated (*72*). There was a reduced enrichment in pathways related to translation machinery of the host, which is supported by studies suggesting that SARS-CoV-2 infection has the ability to reshape translation, splicing, protein homeostasis, and nucleic acid metabolism pathways (*73*, *74*). Thus, the macrophages with detectable SARS-CoV-2 RNA display a transcriptional response associated with up-regulation of inflammatory pathways.

### Adipocyte progenitors in the SVC mount an inflammatory response to SARS-CoV-2 exposure.

**Fig. 6.**
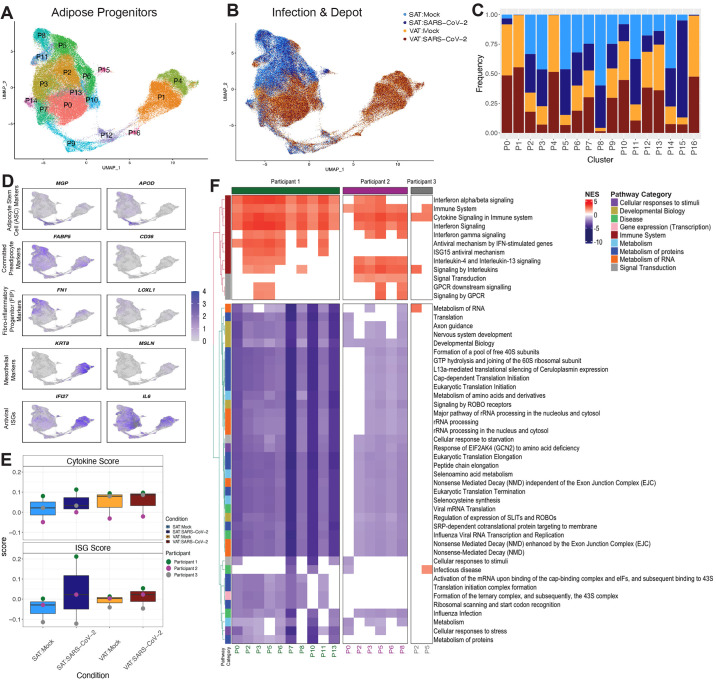
Preadipocytes respond to SARS-CoV-2 exposure. (**A and B**) UMAP embedding is shown for all preadipocytes (n=140,867) colored by cluster (A) and sample and infection type (B) are shown. (**C**) A cell fraction bar plot is shown clustered by sample and infection type within each cluster. (**D**) Feature plots depicting expression of selected markers associated with preadipocyte cell states, cell types, and antiviral ISGs. (**E**) Box plots of average cytokine (top) and ISG (bottom) module scores are shown across the preadipocytes of each participant and depot in both mock and SARS-CoV-2 infection conditions. (**F**) Reactome pathway analysis was performed on the DEGs (padj < 0.1) between SARS-CoV-2-exposed versus mock preadipocytes by participant and cluster within SAT. Pathways that were represented and significant (padj < 0.05) in at least four of the participant-cluster subsets were included. Pathways were clustered by euclidean distance and split by the two major subtrees.

We next evaluated the response of preadipocytes in the SVC to SARS-CoV-2 exposure, as they are responsive to inflammatory mediators such as tumor necrosis factor (TNF)-α or IL-6 (*61*, *75*). We embedded only preadipocytes from samples in Fig. 4A and used unsupervised clustering to identify 17 unique clusters (P0 to P16) (Fig. 6A to C, fig. S14A, data file S8). We did not detect notable amounts of SARS-CoV-2 transcripts in preadipocyte clusters, except for cluster C14 which had only 1.2% of cells with greater than 10 reads. Preadipocytes from SAT and VAT were transcriptionally unique, with 11 of 17 clusters each composed of over 75% of cells derived from only one depot (Fig. 6C). Infection did not shift cluster composition except for cluster P15, which was enriched with preadipocytes from SARS-CoV-2-infected SAT (Fig. 6C). This cluster was inflammatory, with up-regulation of two interferon stimulated genes (ISGs), *IFIT1* and *ISG15* (fig. S14A). The remaining clusters were distinguished by several unique DEGs (fig. S14A,data file S8), including cluster P9, found in both VAT and SAT, which expressed C-X-C motif ligand (*CXCL)14* and *APOD*, markers of adipocyte stem cells (ASCs) (*60*, *61*, *76*)*;* and clusters P1 and P4, VAT clusters that expressed *KRT18* and *MSLN*, markers of mesothelial cells (*60*, *76*, *77*). VAT-predominant clusters demonstrated higher expression of *IFI27* (a marker of SARS-CoV-2 infection in the blood) than other clusters whereas *IL6* expression was high across all preadipocytes in mock-conditions, highlighting the inflammatory nature of preadipocytes at baseline (Fig. 6D).

The preadipocytes derived from SAT had a higher number of DEGs, suggesting a stronger transcriptional response to SARS-CoV-2 exposure, than the paired VAT preadipocytes (fig. S14B). We generated ISG and cytokine gene scores for each participant, infection condition, and depot (Fig. 6E, data file S9). Infection appeared to increase in ISG and cytokine transcripts, with subcutaneous preadipocytes affected more than visceral. In all three participants, the VAT preadipocytes had a higher baseline cytokine expression (Fig. 6E). There was heterogeneity between the individuals, with the greater induction of ISG and cytokine transcripts in participants 1 and 2 compared to participant 3. Although it is difficult to attribute causation with a small number of individuals, participant 3 was administered furosemide and spironolactone (table S1), both drugs shown to exhibit anti-inflammatory effects (*78*–*80*).

We employed a perturbation analysis to examine whether particular preadipocyte clusters from subcutaneous and visceral tissue showed a stronger response to SARS-CoV-2 exposure (*81*, *82*). In preadipocytes from both VAT and SAT, the ASC-like cluster, P9, along with nearby clusters, P16 and P12, showed the lowest perturbation relative to other clusters (fig. S14C and D). Although donors varied in their most highly perturbed clusters, this analysis demonstrated that subcutaneous preadipocytes showed consistently stronger perturbation than visceral preadipocytes upon SARS-CoV-2 infection. Therefore, we focused on the subcutaneous preadipocytes to understand the specific pathways that contribute to the overall inflammatory response. We identified the DEGs between SARS-CoV-2 exposed versus mock preadipocytes by cluster, depot, and participant, and employed gene set enrichment analysis (GSEA) using the REACTOME database to identify relevant pathways to understand the response within each participant and each cluster (Fig. 6F, data files S10 to 13). In all participants, infection induced genes related to cytokine signaling in clusters P5 and P2. In participants 1 and 2, most of the adipose clusters demonstrated enrichment in responses relevant to the immune system, whereas in participant 3, only 2 of 17 clusters showed enrichment for immune-related pathways after infection. Participants 1 and 2 down-regulated pathways related to virus transcription and translation pathways, suggesting that preadipocytes are expressing genes to suppress virus production (Fig. 6F), whereas this did not occur in Participant 3. These data highlight the heterogeneity in preadipocyte responses to exposure to SARS-CoV-2 between participants. A similar analysis was also performed on visceral preadipocytes which demonstrated a similar enrichment for immune response pathways across preadipocyte clusters, with Participant 1 eliciting more changes across their preadipocyte compartment compared to the other two participants (fig. S14D and E). Overall, these data indicate that SARS-CoV-2 infection of SVC, which primarily infects macrophages, can drive inflammatory responses in the neighboring preadipocyte cells.

## DISCUSSION

The SARS-CoV-2 pandemic has substantially impacted global health, with the disease burden disproportionately affecting those suffering from preexisting conditions and socioeconomic inequalities. In particular, obesity increases the risk of severe COVID-19, yet clear mechanisms driving this association remain unknown. Here, we report that SARS-CoV-2 infects human adipose tissue, targeting both mature adipocytes and a subset of adipose tissue macrophages, leading to immune activation and the secretion of inflammatory factors associated with severe COVID-19. This in-depth analysis of adipose tissue susceptibility and inflammatory responses to SARS-CoV-2 infection suggests that adipose tissue could serve as a potential reservoir for SARS-CoV-2 and potentiator of regional or systemic inflammation, possibly contributing to clinical disease in individuals suffering from COVID-19.

Although it is broadly recognized that systemic inflammation induced by SARS-CoV-2 infection can adversely impact organ-specific function (*83*), the extent to which infection of extrapulmonary sites contributes to COVID-19 pathogenesis is less clear. There have been multiple prior reports suggesting that SARS-CoV-2 can infect a range of tissues, including adipose, gut, pancreatic, and brain tissue (*1*, *24*, *26*, *84*–*86*), yet prior studies have generally not assessed whether these sites can contribute to virus propagation by infectious virus quantification, instead generally relying on detection of viral RNA. In our study, several lines of evidence were suggestive of virus replication in adipocytes, including detection of intracytoplasmic SARS-CoV-2 RNA in adipocytes in human autopsy specimens, detection of genomic and subgenomic viral RNA in adipocytes infected in vitro, release of viral RNA into the supernatant of infected cultures, and increasing viral RNA quantification over time. To directly test for infectious virus production, here we used the gold standard plaque assay, providing direct evidence that differentiated adipocytes are permissive to SARS-CoV-2. Thus, adipocytes indeed have the potential to replicate the virus and serve as a SARS-CoV-2 reservoir, as has been similarly proposed for HIV and influenza A virus (*87*–*89*). Importantly, when considered in light of the inflammatory infiltrates we and others (*24*, *26*) observed proximal to SARS-CoV-2-infected cells in autopsy samples, this suggests that infection of adipose tissue could provide an ongoing source of virus replication and inflammation.

Although we show infection of adipocytes with SARS-CoV-2, we were surprised that we did not detect corresponding expression of *ACE2*, the canonical SARS-CoV-2 entry receptor, in fresh adipose tissue. Prior reports of *ACE2* RNA expression in whole adipose tissue relied primarily on bulk transcriptomic approaches and suggested that *ACE2* expression could be influenced by factors including diet, obesity, and COVID-19 (*23*, *24*, *90*–*92*). In freshly isolated mature adipocytes, we only sporadically detected *ACE2* RNA, whereas it was consistently identified in differentiated preadipocytes, consistent with another recent report (*26*). We also did not detect *ACE2* mRNA in scRNA-sequencing data from our own dataset nor in six independent studies that comprised an additional 75 samples (*60*, *61*). Interestingly, a recent report found no correlation between adipose tissue *ACE2* expression with either SARS-CoV-2 infection or body mass index (BMI) (*26*), an in vivo finding mirrored by our in vitro data. Thus, we conclude that *ACE2* RNA expression is variably but rarely detected in freshly isolated tissue, but is induced by the in vitro differentiation process, and that differences between our results and prior studies may reflect different inflammatory conditions, tissue heterogeneity, or technical assay variability.

Importantly, it is ACE2 protein, not RNA, that mediates virus entry. Since protein could be stably expressed on the cell surface even after RNA degradation, we directly assessed ACE2 protein. We did not detect ACE2 by Western blot in mature adipocytes, differentiating adipocytes, or whole adipose tissue, making it highly unlikely that SARS-CoV-2 enters adipocytes through ACE2. We did not further explore the mechanisms leading to the absence of *ACE2* mRNA expression, nor the absence ACE2 protein, in adipocytes undergoing adipogenesis, but possible explanations include overexpression of micro-RNAs targeting *ACE2* (*93*) that can increase in obesity (*94*) or cleavage of ACE2 from the surface of adipocytes by a Disintegrin and Metalloprotease 17 (ADAM17) (*95*–*97*). Overall, this body of evidence suggests that ACE2 is unlikely to play a major role in SARS-CoV-2 infection of adipocytes, which has important implications for the design of therapeutics to block adipose tissue infection. These findings do raise the question of how SARS-CoV-2 enters adipocytes. Expression of alternative SARS-CoV-2 entry receptors like CD147, dipeptidyl peptidase 4 (DPP4), and neuropilin 1(NRP1) have been reported in adipocytes and their expression is increased by obesity and decreased by anti-inflammatory myokines (*98*). A recent study also detected higher expression of *NRP1* and *FURIN* (additional proposed SARS-CoV-2 viral entry factors) than of *ACE2* and *TMPRSS2* in murine and human adipocytes (*25*). Future work should focus on identifying the entry receptor(s) involved in SARS-CoV-2 infection of adipocytes to inform therapeutic targets.

An important finding of our study is that adipocytes are not the only target of SARS-CoV-2 in adipose tissue, as we also detect SARS-CoV-2 RNA in a subset of inflammatory adipose tissue-resident macrophages. This finding is consistent with recent reports that SARS-CoV-2 can infect macrophages from the lung and secondary lymphoid organs, driving inflammatory pathology (*38*, *99*–*104*). In our study, we did not detect infectious virus release, suggesting abortive infection of adipose tissue macrophages. In other tissues, whether macrophages can be productively infected remains debated. Two studies show productive infection of human alveolar macrophages and MDMs (*103*, *104*). However, numerous other studies report abortive infection of monocytes, macrophages, and MDM, hypothesizing that low ACE2 expression or pyroptosis prior to virus production and release lead to abortive infection (*38*, *39*, *105*–*107*). Interestingly, the entry into the productively infected alveolar macrophages was ACE2-dependent, whereas in our study and another recent report (*39*), entry into macrophages is ACE2-independent and leads to abortive infection. This further supports the idea that entry mechanism could influence the ability of the virus to replicate in macrophages. It is also important to consider that there is heterogeneity among macrophage populations. Transcriptional profiling enabled us to identify two unique subsets of adipose tissue-resident macrophages, neither of which fell into a classical M1 versus M2 classification, consistent with reports that macrophages in adipose tissue exhibit unique phenotypes (*39*). One of these subsets, an inflammatory cluster characterized by high expression *IL1B* and *CCL3*, was preferentially infected. This parallels recently reported results of heterogeneity in macrophage susceptibility to SARS-CoV-2 in the lung. One study showed that a subset of activated interstitial macrophages are a predominant target of SARS-CoV-2 in the human lung and another demonstrated differences in the susceptibility of M1 alveolar macrophages versus M2 alveolar macrophages (*108*). Overall, it seems likely that distinct macrophage subsets or tissue sites may differ in their permissiveness to SARS-CoV-2. Further investigation into drivers of increased macrophage susceptibility to SARS-CoV-2 and whether these pathways are influenced by obesity may help to better define the mechanistic basis for the association between obesity and COVID-19 severity.

Importantly, we found that infection of adipose tissue stromal vascular cells drives an inflammatory response, characterized by increased secretion of cytokines and chemokines associated with COVID-19 severity, including IP-10, PDGFAA, PDGFAB/BB, IL-4, macrophage migration inhibitory factor (MIF), vascular endothelial growth factor A (VEGF), and macrophage colony-stimulating factor 1 (MCSF) (*43*, *45*, *109*–*116*), and the induction of ISGs within both the infected macrophages and within the generally uninfected preadipocytes. Notably, this inflammatory response was heterogeneous at both the cellular and participant level; of the 17 identified preadipocyte clusters in VAT and SAT, ASC-like clusters demonstrated the most subdued transcriptional response to infection, and participants differed in the magnitude of the infection-induced inflammatory response, which is difficult to attribute to any single comorbidity or underlying inflammatory state. Regardless, the induction of an inflammatory response in stromal vascular cells has important implications for COVID-19 because adipose tissue secretion of proinflammatory mediators could contribute to systemic inflammation (*117*).

Our study has several limitations, including a small number of individuals and samples in some assays due to the difficulty in obtaining human tissue and the challenges of performing single cell sequencing and other assays with fragile adipose tissue in the biosafety level 3 (BSL3) environment. Additionally, it is possible that our detection of plaques following infection of differentiated adipocytes was a result of retained virus inoculum; however, the fact that we extensively washed input virus after the one hour exposure and never detected plaques following infection of stromal vascular cells or differentiated macrophages makes this unlikely. All experiments were performed with the WA-01 strain of SARS-CoV-2, and it will be important for future studies to evaluate other SARS-CoV-2 variants. For our in vitro studies, the tissue donors were obese, and it will be important for future studies to evaluate responses to infection in adipose tissue from lean individuals.

Overall, our data are consistent with a model in which SARS-CoV-2 infection of adipose tissue could contribute to COVID-19 severity through two mechanisms which could act in a feed-forward loop: i) amplification of virus within adipocytes and ii) local and systemic inflammation primarily instigated by inflammatory responses from abortively infected adipose tissue-resident macrophages. It is therefore critical that future studies explore both how to block adipocyte and macrophage infection and whether inhibiting inflammatory responses within the adipose tissue could reduce COVID-19 disease severity. Inhibition of adipose tissue inflammation is already being explored as a treatment for metabolic disease during obesity (*109*–*115*), and extension of such strategies to COVID-19 could be beneficial. For instance, salicylate, a cyclooxygenase inhibitor, reduces adipose tissue inflammation in obese individuals and has been proposed as a therapeutic strategy in patients with COVID-19 due to its anti-inflammatory properties and antiviral activity against both DNA and RNA viruses (*109*–*115*). Additionally, if adipocytes constitute a reservoir for viral infection, this ongoing source of replication could fuel long COVID syndrome, a possibility that also must be explored in future studies.

## MATERIALS AND METHODS

**Study design:** The aim of this study was to determine if human adipose tissue is permissive to SARS-CoV-2 infection. We obtained adipose tissue from individuals after consent and exposed these samples to SARS-CoV-2 in vitro and measured viral entry, replication, and inflammatory pathways by flow cytometry, RTqPCR, scRNA-seq, and Luminex. All analyses were performed in an unbiased fashion. Formalin-fixed and paraffin embedded lung, kidney, adipose and heart tissue from COVID-19 autopsy samples were evaluated for viral RNA. The n value was not controlled and was dependent on availability of samples.

**Statistical analysis:** Raw, individual level data are presented in data file S14. GraphPad Prism version 9.1.0 (216) and R (4.0.4) were used for statistical analysis. When comparing more than two groups a two-way ANOVA, multiple comparisons using the Sidak correction was performed. In Luminex analysis, a paired Wilcoxon signed rank test with desired false discovery rate of 10% was performed. Data bars are always presented as mean ± s.e.m.

## Supplementary Materials

### This PDF file includes:

Materials and Methods Figs. S1 to S14 Tables s1 and S2 References (*118*–*123*)

### Other Supplementary Material for this manuscript includes the following:

MDAR Reproducibility Checklist
