# Supplementary material for: SARS-CoV-2 infection drives an inflammatory response in human adipose tissue through infection of adipocytes and macrophages
Source: Sci Transl Med. 2022 Sep 22:eabm9151. doi: 10.1126/scitranslmed.abm9151 (PMC9529056; doi:10.1126/scitranslmed.abm9151)
Supplement: Supplementary file 1 — Materials and Methods Figs. S1 to S14 Tables s1 and S2 References ( 118 – 123 ) [file scitranslmed.abm9151_sm.pdf]

Science Translational Medicine
AAAS

# Supplementary Materials for

## SARS-CoV-2 infection drives an inflammatory response in human adipose tissue through infection of adipocytes and macrophages

Giovanny J. Martínez-Colón *et al.*

Corresponding authors: Tracey L. McLaughlin, tmclaugh@stanford.edu; Catherine A. Blish, cblish@stanford.edu

DOI: 10.1126/scitranslmed.abm9151

**The PDF file includes:**

Materials and Methods
Figs. S1 to S14
Tables S1 and S2
References (*118*–*123*)

**Other Supplementary Material for this manuscript includes the following:**

MDAR Reproducibility Checklist
Data files S1 to S14

**Title:** SARS-CoV-2 infection drives an inflammatory response in human adipose tissue through infection of adipocytes and macrophages


**Authors:**

Giovanny J. Martínez-Colón[1]†, Kalani Ratnasiri[2]†, Heping Chen[1], Sizun Jiang[5,9], Elizabeth Zanley[1], Arjun Rustagi[1], Renu Verma[1], Han Chen[5], Jason R. Andrews[1], Kirsten D. Mertz[10], Alexandar Tzankov[8], Dan Azagury[3], Jack Boyd[4], Garry P. Nolan[5], Christian M. Schürch[7], Matthias S. Matter[8], Catherine A. Blish[1,2,6]†*, Tracey L. McLaughlin[1]†*

**Supplementary Materials**

Materials and Methods

fig. S1: RNA-ISH assay controls for lung and adipose tissue.

fig. S2: In vitro differentiated adipocytes harbor SARS-CoV-2 RNA after exposure to SARS-CoV-2.

fig. S3: Adipocytes support SARS-CoV-2 infection.

fig. S4: No ACE2 protein detection in adipose tissue cells.

fig. S5: No plaque forming units were observed in cultures using supernatants from SVC or monocyte-derived macrophages exposed to SARS-CoV-2.

fig. S6: Gating strategy of SVC in human adipose tissue.

fig. S7: Elevated *IL6* gene expression in SVC post in vitro SARS-CoV-2 infection.

fig. S8: Increased interferon related genes in adipose tissue post-SARS-CoV-2 infection.

fig. S9: Infection, depot and participant breakdown by cluster annotation.

fig. S10: Distribution of SARS-CoV-2 transcripts across all cells.

fig. S11: Limited ACE2 protein detection in SVC from SAT and VAT.

fig. S12: *ACE2* expression across public human adipose single-cell RNA-sequencing datasets.

fig. S13: C2- and C12-macrophages are distinctly different upon mock and SARS-CoV-2 infection.

fig. S14: Characterization of preadipocytes across VAT and SAT.

Table S1. Adipose tissue participant's demographic, medical information, and sample use.

Table S2. Antibodies and reagents used in flow cytometry

## SUPPLEMENTARY MATERIAL

### Materials and Methods

**Participants:** Study participants were recruited from the Adult Bariatric Surgery and Cardiothoracic Surgery clinics at Stanford University Medical Center during the preoperative visit. Eligibility requirements included age greater than 25 years, and exclusion criteria included chronic inflammatory conditions, pregnancy or lactation, use of weight loss medications, and current diagnosis of COVID-19 by polymerase chain reaction (PCR) test. Serological assessment for previous infection was not performed. This was an observational study with the protocols approved by the Stanford Institutional Review Board and registered on clinicaltrails.gov (Stanford IRB approval/ trial registration number: IRB-40196/NCT04708535 and IRB-52647/NCT04167761) and all participants gave written, informed consent. Tissue samples from COVID-19 deceased patients were obtained from University Hospital Tübingen, Tübingen, Germany, or from University Hospital Basel or Cantonal Hospital Baselland, Switzerland. Samples were de-identified and SARS-CoV-2 infected tissue was obtained during autopsy and processed as previously described (32). The use of SARS-CoV-2 infected tissue was approved by the ethics commission of Northern Switzerland (EKNZ; study ID: 2020-00969). All COVID-19 patients or their relatives consented to the use of tissue for research purposes.

**Preparation, isolation, and differentiation of adipose tissue:** On the day of bariatric surgery, approximately 2 to 3g each of subcutaneous abdominal (SAT) and omental visceral adipose (VAT) tissue was harvested intraoperatively and immediately processed. For cardiothoracic surgery patients, 2g of pericardial adipose tissue (PAT), 1g of epicardial adipose tissue (EAT), and 1 to 2g of SAT chest wall was obtained intraoperatively and immediately processed. Tissue was subject to collagenase digestion for separation of mature adipocytes (MA) and stromal vascular cells (SVC), and for differentiation of preadipocytes as previously described (118).

**Adipose Tissue Processing:** Adipose tissue samples were minced and then digested by collagenase I (1 mg/mL) (Worthington Biochem. Corp) at 37ºC for 60 minutes, in Krebs-Ringer solution HEPES-buffered (KRBH, Alfa Aesar) containing bovine serum albumin (BSA; 2.5%, Sigma-Aldrich), adenosine (250 µM, Acros Organics), and penicillin/streptomycin (P/S; 1%, Thermo Fisher Scientific), then filtered through a 500-µm nylon mesh, followed by separation of MA and SVC. MA were utilized immediately; SVC was collected by centrifugation at 500 x g for 5 minutes at room temperature. The SVC pellet was incubated in erythrocyte lysis buffer (ACK erythrocyte lysis buffer, Invitrogen) for 10 minutes at 37ºC, followed by centrifugation as above. The cell pellet was resuspended in Hank's Balanced Salt Solution (HBSS; HyClone, SH3026802) with 2% BSA (Sigma-Aldrich) and centrifuged for another 5 minutes at 500 x g at room temperature. The SVC pellets were resuspended in growth medium consisting of Dulbecco’s Modified Eagle’s Medium/Ham’s Nutrient Mixture F-12 (DMEM/F-12; HyClone, SH3002302), 10% fetal bovine serum (FBS, Corning), and 1% P/S), filtered through a 75-µm cell strainer, and cultured for expansion or immediate use.

**Differentiation of human preadipocytes:** As previously described (118), isolated SVC was expanded in DMEM/F12 containing FBS (10%) and P/S (1%), split to expand, and cultured until confluence. When cells reached 100% confluence, differentiation was induced using differentiation media, DM-2 (Zenbio, Inc) consisting of insulin, dexamethasone and isobutylmethylxanthine (IBMX) and pioglitazone supplemented with 10% FBS. After 3 days, the media was changed to adipocyte maintenance media, AM-1 (Zenbio, Inc) containing only insulin, dexamethasone in DMEM/F12 supplemented with 10% FBS. The cells were then left to differentiate for 3, 6, 12, and 14 days, with culture medium (AM-1) changed every 3 days. Day 0 preadipocytes were collected at time of confluence, just before adipogenesis induction. During differentiation, cells were collected at all the other time points and utilized immediately for experiments as described. Adipogenesis was confirmed by oil droplet formation and by increased expression of fatty acid-binding protein 4 gene, *FABP4*.

**Differentiation of human monocyte-derived macrophages (MDMs):** Production of MDMs was achieved by first isolating monocytes from peripheral blood mononuclear cells (PBMCs) using the pan monocyte isolation kit (Miltenyi Biotec, 130-096-537) following manufacturer's recommendation. Monocytes were then cultured at a density of $1x10^6$ cells per ml in culture media (10% FBS, DMEM, and P/S) containing 50ng/ml of macrophage colony stimulating factor (MCSF) (Gibco, PHC9501) for 7 days. Media was changed every 2 to 3 days and supplemented with 50 ng/mL MCSF.

**Virus and cell lines:** The USA WA1/2020 strain of SARS-CoV-2 was obtained from the Biodefense and Emerging Infections Research Resources Repository (BEI Resources), passaged

in VeroE6 cells, and titered by Avicel (FMC Biopolymer) overlay plaque assay on VeroE6 cells. Passage 3 virus was used for all infections. The virus was deep sequenced and aligned to confirm no substantial mutations from the GenBank sequence. VeroE6 cells were obtained from the American Type Culture Collection (ATCC) and were mycoplasma free. VeroE6 cells expressing transmembrane protease, serine 2 (VeroE6/TMPRSS2) were obtained from the Japanese Collection of Research Bioresources (JCRB) cell bank. A549-ACE2 cells were a gift from Ralf Bartenschlager and were mycoplasma free.

**SARS-CoV-2 infection of differentiated adipocytes, SVC, MDMs, and A549-ACE2:** Cells were seeded a day before infection by culturing $4x10^5$ to $1x10^6$ cells per well in a 6-well plate (Corning). On the day of infection, SVC was centrifuged at 500 x g for 5 minutes, and washed with infection media (DMEM, 2% FBS, and 1% P/S). Adherent differentiated adipocytes and A549 cells expressing human angiotensin converting enzyme 2 (A549-ACE2 cells) were washed with infection media. A549-ACE2 cells were cultured under the presence of 623µg/ml of Geneticin (Thermo Fisher Scientific; 10121035) for selection of ACE2-expressing cells. Viral infection was performed with SARS-CoV-2 (2019-nCOV/USA-WA1/2020) at a multiplicity of infection (MOI) of 1 for 1 hour while gently rocking. Cells were then washed and cultured in culture media (DMEM, 10%FBS, and 1% P/S) at 37ºC with 5%$CO_2$ under BSL3 containment.

**SARS-CoV-2 infections of MA:** MA media was changed into infection media by penetrating the fat tissue with a 22-gauge polytetrafluoroethylene (PTFE) (Grainger) blunt needle attached to a 3cc syringe (Grainger), or by gently transferring the fat with a wide manually cut p100 tip into a 5ml conical collection tube containing 2mls of warm media. Viral infection was performed

with the WA/01 strain of SARS-COV-2 (2019-nCOV/USA-WA1/2020) at a MOI of 1 by gently adding virus to the top of the floating cells under BSL3 containment. MA was incubated for 1 hour while gently rocking at 37ºC with 5%$CO_2$. Media was then removed using as mentioned above prior to adding culture media (DMEM, 10%FBS, and 1% P/S) and incubating at 37ºC with 5%$CO_2$.

**Flow Cytometry:** Cells were collected using Trypsin-EDTA (Thermo Fisher Scientific, 25200072) for 5 to 10minutes at 37ºC and 5% $CO_2$. Cells were then centrifuged at 500 x g for 5 minutes, and washed with PBS (Thermo Fisher Scientific, 10010023) and transferred into a 96-well plate; cells were then washed twice more with FACS buffer consisting of PBS plus 2% FBS and 5mM EDTA (Hoefer, GR123). Cells were incubated in Zombie aqua (1:100, BioLegend, 423101) cell viability dye (BioLegend, 423102) at room temperature for 20 minutes, washed twice, incubated at room temperature with Fc block (BioLegend, 422302) for 5 minutes, and surface stained for 30 minutes at 4ºC. Surface staining contained antibodies for CD45, CD3, CD14, CD19, CD11c, CD34, and CD31 (antibody details in table S2). In fig. S4, surface staining also contained antibodies against ACE2 or isotype control (antibody details in table S2). After surface staining, cells were washed twice with FACS buffer before fixation with 4% paraformaldehyde (PFA) (Electron Microscopy Sciences, 15710) for 1 hour at 4ºC; cells were then permeabilized (eBioscience, 00-8333-56) for 10 minutes at room temperature before intracellular staining for 45 minutes at 4ºC. Intracellular staining contained an anti-SARS-CoV-2 N protein antibody (antibody details in table S2). After intracellular staining, cells were washed with PBS, and diluted in 1% PFA and PBS before analyzing on a Cytek Aurora. FCS files were collected and analyzed using FlowJo. Gating was done as shown in fig. S6 and 11. Pictures of

SVC for fig. S6 were taken with the use of a EVOS XL core cell imaging system (Thermo Fisher Scientific) with an objective of 10x.

**RNA isolation and quantification:** At the time of collection, cells were washed with phosphate-buffered saline. Samples were then incubated with TRIzol LS (Thermo Fisher Scientific) reagent for 15 to 20 minutes for cell lysis, virus inactivation, and RNA extraction. RNA was resuspended in water and quantified by absorbance (260/280) using a NanoDrop spectrophotometer (Thermo Fisher Scientific).

**Genomic and subgenomic absolute gene quantification by real time quantitative polymerase chain reaction (RTqPCR):** 5µl of the total RNA was used for 1-step RTqPCR. Genomic *N* gene quantification was done with the use of CDC qualified primers and probes amplifying the *N1* region, n2019-nCoV (Biosearch technologies, KIT-NCOV-PP1-1000). For subgenomic *N* gene quantification, *E* gene sgRNA forward primer for SARS-CoV-2 leader sequence was combined with CDC *N1* gene reverse primer and probe to detect *N* gene sgRNA as previously shown (119, 120). RNA and primers were mixed with the TaqPath 1-step RTqPCR master mix (Applied Biosystems, A15299). A standard curve for Ct values and genome copy numbers was obtained using pET21b+ plasmid with the *N* gene inserts. All the samples were analyzed in technical duplicates. The Ct cutoff for positive samples was below 38 with amplification observed in both duplicates. The samples were analyzed on a StepOnePlus real time PCR machine (Applied Biosystems) using the following parameters: (stage 1) 10 minutes at 50ºC for reverse transcription, followed by (stage 2) 3 minutes at 95ºC for initial denaturation and (stage 3) 40 cycles of 10 seconds at 95ºC, 15 seconds at 56ºC, and 5 seconds at 72ºC.

**RTqPCR for host transcripts:** RNA was diluted in ultra-pure water (Invitrogen, 10977023) and mixed with TaqPath 1-step RTqPCR master mix (Applied Biosystems, A15299) and primers for SARS-CoV-2 *N* gene (Biosearch technologies; n2019-nCoV KIT-NCOV-PP1-1000), "Full length" *ACE2* (Thermo Fisher Scientific, Hs01085335_m1), "total" *ACE2* (Thermo Fisher Scientific, Hs01085333), *IL6* (Thermo Fisher Scientific, Hs00174131_m1), *IFNA1* (Thermo Fisher Scientific, Hs04189288_g1), *IFNB1* (Thermo Fisher Scientific, Hs01077958_s1), *ISG15* (Thermo Fisher Scientific, Hs01921425_s1), *IFI27* (Thermo Fisher Scientific, Hs01086373_g1),and *IER3* (Thermo Fisher Scientific, Hs00174674_m1) . *ACE2* isoform's forward (GTGAGAGCCTTAGGTTGGATTC), reverse (TAAGGATCCTCCCTCCTTTGT) and probe (FAM-TCATTGAGGAGAGCTCTGAGGCAGA-BHQ) were made using beta-cyanoethyl phosphoramidite chemistry at Stanford's Protein and Nucleic Acid Facility. All sequences are Sequence 5'→3'. Values presented as ΔCT, were obtained using the endogenous control eukaryotic *18S* rRNA (Thermo Scientific, 4352930E). Values presented as ΔΔCT were obtained using *18S* rRNA as endogenous control and mock samples as the calibrator control. The samples were analyzed on a QuantStudio 3 (Applied Biosystems) with the following parameters: 2 minutes at 25ºC for uracil-DNA glycosylase (UNG) incubation, 15 minutes at 50ºC for reverse transcription, 2 minutes at 95ºC for polymerase activation, and 40 cycles of 15 seconds at 95ºC and 30 seconds of 60ºC for amplification.

**ACE2 protein detection by western blotting:** Human whole adipose tissue protein cell lysate was commercially obtained (NB820-59170). Cell lysates from A549 cells, A549-ACE2 cells, differentiated adipocytes, and mature adipocytes were obtained fresh by cell lysis using RIPA

buffer (Thermo Fisher Scientific, 89900) supplemented with Halt protease inhibitor cocktail (Thermo Fisher Scientific, 78420), and EDTA as recommended by the manufacturer. Briefly, cells were washed multiple times with cold PBS, followed by resuspension in ice-cold RIPA-protease-EDTA buffer. Cells were then incubated for 15 minutes on ice prior to centrifugation at 14,000 x g for 15 minutes at 4ºC to remove cellular debris. Cell lysates were mixed with Laemmli sample buffer (Bio-Rad, 1610727) containing 5% of 2-mercapthoeathanol (Millipore, M3148) and heated for 15 minutes at 95 to 100ºC. Protein electrophoresis was performed with NuPAGE 4 to 12%, Bis-Tris protein gels using the mini gel tank system (Invitrogen, A25977) filled with NuPAGE MOPS SDS running buffer (Invitrogen, NP0001). Cell lysates in PAGE gel were transferred to polyvinylidene fluoride (PVDF) membranes using the iBlot 2 dry blotting system (Invitrogen). Membranes were then blocked with 5% BSA (Millipore, A7979) in Tris-buffered saline (TBS). Membranes were incubated overnight at 4ºC with rabbit anti-human ACE2 antibodies raised against sequences near the N-terminus (Abcam, ab108252, 1:5000) or the C-terminus (Abcam, ab15348, 1:5000) as well as a loading control, goat anti-human GAPDH (Invitrogen, PA19046, 1:10000). Membranes were washed with TBS containing 0.05% Tween20 (Bio-Rad, 1662404) prior to incubation with fluorescent secondary antibodies, donkey anti-Rabbit IgG (IRDye-800CW) (LI-COR, 926-32213, 1:15000) and donkey Anti-Goat IgG (IRDye-680RD) (LI-COR, 926-68074, 1:15000). Blots were read using the LI-COR Odyssey Near-IR scanner. Images were analyzed using the Empiria studio software.

**SARS-CoV-2 plaque assay:** VeroE6 cells encoding the human transmembrane serine protease 2 (TMPRSS2),VeroE6/TMPRSS2, were obtained from the Japanese Collection of Research Biosources Cell Bank (JCRB1819) and used for plaque quantification. Briefly,

VeroE6/TMPRSS2 were seeded in 12-well plates until full cellular monolayer was achieved. Cells were washed multiple times with PBS followed by inoculation with supernatants or viral stocks. An overlay mixture of 1.2% Avicel (Chemical book, CB6954460) with minimum-essential media (MEM) (Thermo Fisher Scientific, 11935046) supplemented with 0.2% BSA (Sigma-Aldrich, A7979), 2mM L-Glutamine (Thermo Fisher Scientific, SH30034), 1% P/S, 0.125% sodium bicarbonate (Gibco, 10378016), and 0.01M HEPES (Gibco, 15630-080) was added to the cells. Cells were then incubated for 72 hours at 37°C and 5%$CO_2$ prior to removal of avicel overlay and fixation with 70% Ethanol. Wells were then stained with 0.3% Crystal Violet (Sigma-Aldrich, V5265).

**SARS-CoV-2 detection in autopsies:** Autopsy samples were prepared as previously described (121). Briefly, RNA was isolated from formalin-fixed and paraffin embedded tissue with the use of Maxwell RSC RNA FFPE (Promega) according to manufacturer recommendations. RTqPCR was performed using the TaqMan 2019-nCoV control kit v1 (Thermo Fisher Scientific, A47533) to target the three viral genes: *ORF1ab*, *S*, and *N* genes, and the human *RPPH1* gene. A Ct value below 37 in at least two out of three viral genomic regions was considered positive per manufacturer's recommendation. A case was considered negative if Ct values were above 40. Values between 37 and 40 were considered undetermined and the assay was repeated. Samples were always run in duplicates. RNA-ISH was used to detect SARS-CoV-2 spike mRNA in tissue samples, described below.

**RNA in situ hybridization (RNA-ISH):** For deparaffinization, slides were baked at 70 °C for 1 hour in a temperature-controlled oven, then immersed in fresh xylene twice for 5 minutes each.

Rehydration was performed using a Leica ST4020 Linear Stainer (Leica Biosystems) programmed to three dips per wash for 180 seconds each with the following buffers: xylene x 3, 100% ethanol x 2, 95% ethanol x 2, 80% ethanol x 1, 70% ethanol x 1, and ddH2O x 3. Heat-induced epitope retrieval was subsequently performed in a Lab Vision PT module (Thermo Fisher Scientific) using the Dako Target Retrieval Solution, pH 9 (DAKO Agilent, S236784-2) at 97 °C for 10 minutes, followed by controlled cooling down to 65 °C. Slides were removed from the PT module and further cooled to room temperature for 30 minutes before rinsing briefly in sterile, nuclease-free water ($ddH_2O$) twice. A 15 minute hydrogen peroxide block was subsequently performed at 40 °C (Bio-Techne, 322335). Slides were then washed twice for 2 minutes each in $ddH_2O$ before an overnight hybridization at 40 °C with probes against SARS-CoV-2 spike mRNA (Bio-Techne, 848561). Amplification of the ISH probes were performed the next day according to the manufacturer's protocol (Bio-Techne, 322350). Slides were then dried, mounted, and subsequently scanned using an Aperio slide scanner at the Stanford School of Medicine Histology Core.

**Sample preparation for Luminex:** Supernatants from mock or infected SVC were collected after 24 hpi into low-binding protein collection tubes. Supernatants from mature adipocytes were collected by first removing floating mature adipocytes with a cut wide pipette tip and pipetting remaining media into low-protein binding collection tubes. Supernatants were stored at -80ºC. To remove samples from BSL3 containment, supernatants were thawed and mixed with 10% TritonX-100 (Sigma-Aldrich, T9284) for a final concentration of 1% TritonX-100 and incubated for 20 minutes at room temperature for viral inactivation. Supernatants were then removed from

BSL3 and transferred to a low-binding protein 96 well plate for 80plex Luminex by the Human Immune Monitoring Center (HIMC) at Stanford University.

**Single-cell RNA sequencing (scRNA-seq)**: The gel beads-in-emulsion (GEM) single cell 3' kit v3.1 dual index kit (10X Genomics) was used following manufacturer's recommendations with slight modifications. Briefly, SVC from SAT and VAT was left untreated or infected with SARS-CoV-2 as described above. At 24hpi, cells were collected, washed, and diluted at a density of $1x10^3$ cells per µl in cold Dulbecco's Modified Eagle Medium (Life technologies; 11885-092) supplemented with 10% fetal bovine serum (Corning, MT35016CV). 10,000 cells per lane were loaded onto a Chromium Controller in the Biosafety Cabinet (BSC) per manufacturer's instructions. Following GEM creation, samples were transferred into PCR tube strips prior to transferring into a PTC-200 thermocycler (MJ Research) for reverse transcription. The reverse transcription parameters were the following: 45 minutes at 53ºC, followed by 5 minutes at 85ºC, then 15 minutes at 60ºC and finally samples were kept at 4ºC. Barcoded cDNA was removed to BSL2 and sequencing libraries were prepared per manufacturer's recommendation, with a TapeStation 4200 (Agilent) used for quality control. Libraries were pooled for a final concentration of 10nM and sequenced on a NovaSeq S2 (Illumina) at the Chan Zuckerberg Biohub (San Francisco).

**Alignment and preprocessing of scRNA-seq data:** The quality of the raw FASTQ data was examined with FASTQC and then aligned (cell ranger count) to a custom genome including human genome (hg38) and the complete genome sequence of SARS-CoV-2 (2019-nCOV/USA-WA1/2020) (GenBank: MN985325.1) using the "Cell Ranger" software package v6.0.0 (10x

Genomics). R (4.0.4) was used for all downstream analyses. Resulting filtered feature-barcode-matrices were processed using the R package Seurat (v4.0.0). Briefly, count matrices were merged and loaded into Seurat with SARS-CoV-2 counts removed and appended to the metadata. All genes represented in fewer than 10 cells were excluded from downstream analysis. Cells were filtered based on the following criteria: less than 200 distinct genes, less than 100 unique molecular identifiers (UMIs), and greater than 15% UMIs from mitochondrial genes. Each batch was then individually normalized using the "SCTransform" function that included regression for percent mitochondria. Integration features were then calculated using the "SelectIntegrationFeatures" function and passed into "VariableFeatures" of the merged object to maintain the repeatedly variable features across each dataset. Within each batch and condition (tissue and infection status), cells identified as doublets by both "DoubletFinder" (v3) (using pN = 0.25, pK = 0.09, PCs = 1:50, anticipated collision rate = 10%) and scds (top 10% of cells ranked by hybrid scores) were removed (n = 3,165 cells) from the analysis. After applying these filtering steps, the dataset contained 198,759 high-quality cells. Principal component (PC) analysis (PCA) was performed on the data. The resulting data were corrected for batch effects using the "Harmony" package (59) with the top 50 PCs. Uniform Manifold Approximation and Projection for Dimension Reduction (UMAP) coordinate generation and clustering were performed using the "RunUMAP", "FindNeighbors", and "FindClusters" (resolution = 0.6) functions in Seurat with PCs 1 to 50. Manual annotation of each cellular cluster was performed by finding the differentially expressed genes using Seurat's "FindAllMarkers" function with default Wilcoxon rank-sum test and comparing those markers with known cell type-specific genes from previous datasets (60, 61). "FindMarkers" function using the MAST algorithm

(latent.vars = ‘participant’) based on a Bonferroni-adjusted $P < 0.05$ and a log2FC > 0.25 was used for targeted differential gene expression analysis.

**scRNA-seq analyses:** Gene ontology and Kyoto Encyclopedia of Genes and Genomes (KEGG) pathway analysis was performed using R package stringdb (122). REACTOME GSEA was performed using the R package fgsea (123), considering ranked gene lists. The Seurat function AddModuleScore() was used to score individual cells by expression of either a list of genes relating to ISGs or cytokines. This function generates an average module score by calculating the mean expression of each gene in the module corrected for expression of a random set of similarly expressing genes. Gene lists used to define each module are defined in data file S9. Heatmaps were generated using ComplexHeatmap, Seurat and pheatmap packages, Violin plots were generated using ggplot2, and dotplots were generated using the Seurat packages in R. Reanalysis of public scRNA-seq datasets was done by first identifying human adipose tissue datasets uploaded to Gene Expression Omnibus (GEO) which were a total of six datasets (GSE155960, GSE129363, GSE196518, GSE156110, GSE176067, GSE176171)(60, 61, 65). The count matrices and metadata from each identified dataset was downloaded, loaded into Seurat with no filtration on cell quality, and Seurat’s “FeaturePlot” function was performed on all cells on the basis of *MGP*, *PTPRP*, and *ACE2* expression. A Zenodo repository for code used for analysis and figures is available at DOI: 10.5281/zenodo.7038876 and sequencing data is available under the NCBI Gene Expression Omnibus accession number GSE208034.

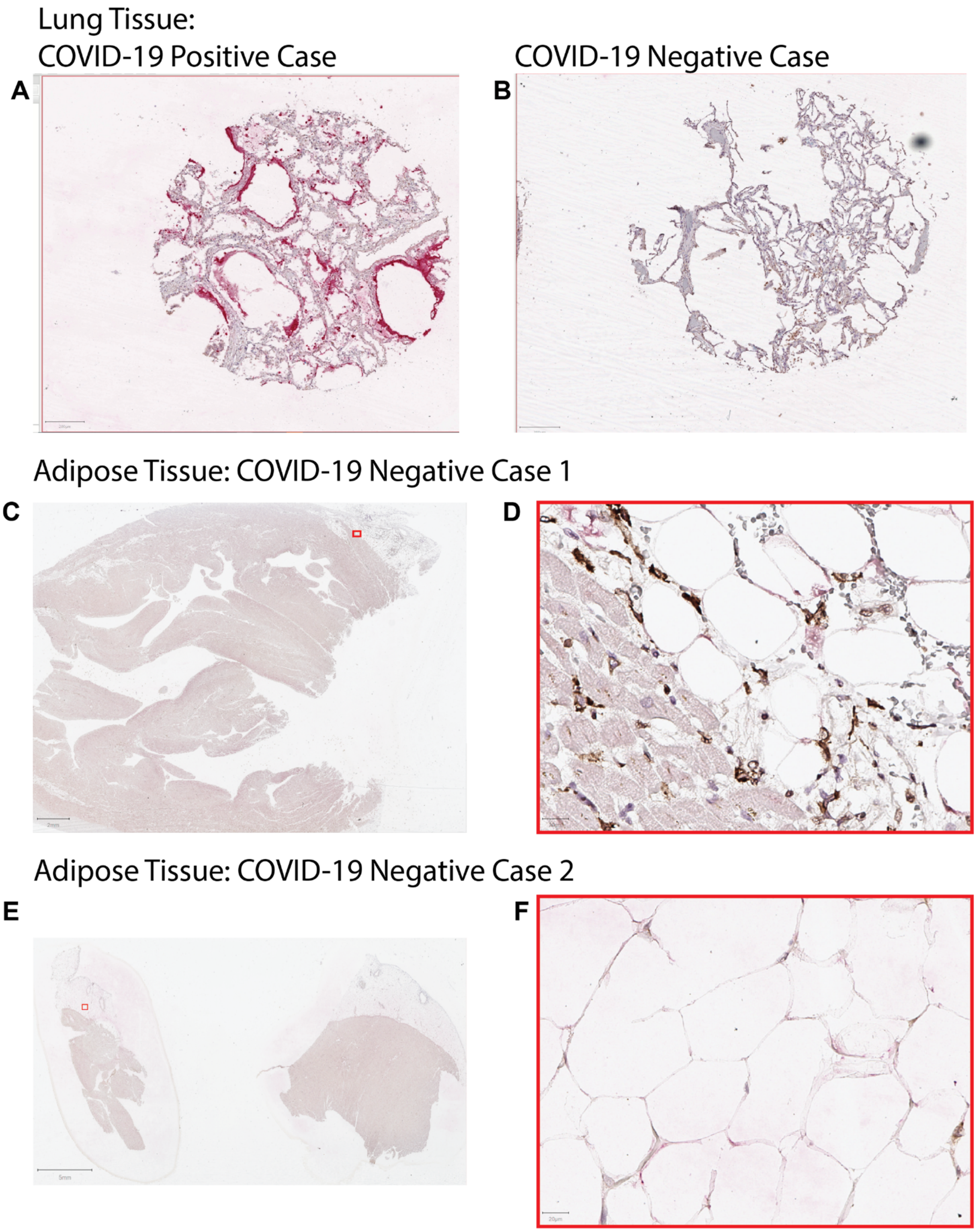


**Fig. S1. RNA-ISH assay controls for lung and adipose tissue**. Assays were performed using probes against SARS-CoV-2 Spike mRNA (red staining). (**A and B**) Lung histology overviews of COVID-19 positive (A) and negative (B) cases. (**C to F**) Overview of the adipose tissue from two negative cases (C and E) and magnified view (20µm) (D and F) of the indicated region. Scale bars in (A and B) indicate 200µM, scale bar in (C) indicate 2mm, scale bar in (D and F) indicate 20µm, and scale bar in (E) indicates 5mm. Brown staining in (D) indicates CD68. All cases are independent.

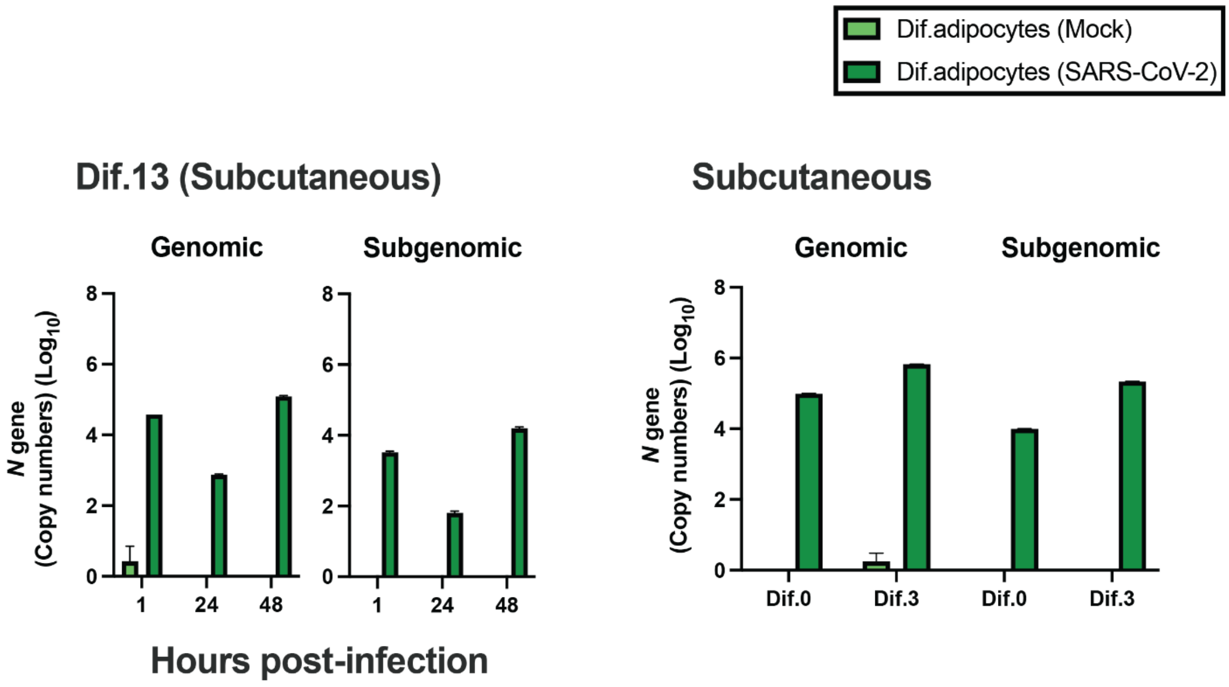


**Fig. S2. In vitro differentiated adipocytes harbor SARS-CoV-2 RNA after exposure to SARS-CoV-2.** Differentiated (Dif.) adipocytes were infected or left untreated (mock) for 24 hours with SARS-CoV-2 (USA-WA1/2020) at a MOI of 1. Measurements of genomic and subgenomic SARS-CoV-2 *N* gene copy numbers in mock and infected adipocytes differentiated from preadipocytes of subcutaneous adipose tissue with selection media for 13 days (infected for 1, 24, and 48 hours) (left) and 0 or 3 days (infected for 24 hours) (right); data was obtained by absolute gene quantification using 1-step RTqPCR TaqMan and reported as log10 values of genome copy numbers. Data presented as ± mean s.e.m.

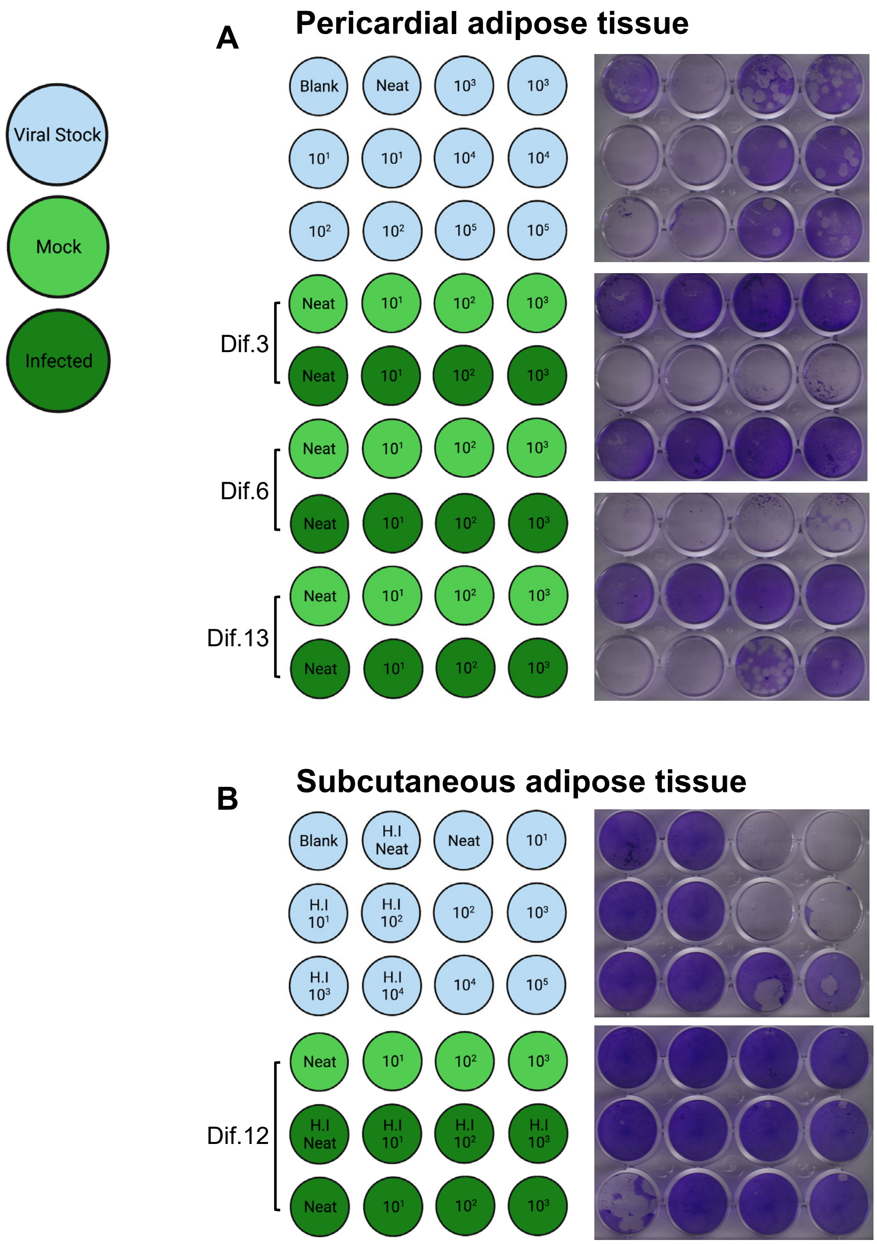


**Fig. S3. Adipocytes support SARS-CoV-2 infection. (A and B)** Adipocytes were obtained by in vitro differentiation of adipocyte precursor cells from fresh stromal vascular cells collected post-collagenase digestion from either (A) pericardial or (B) subcutaneous adipose tissue. Cells were infected (dark green), or mock infected (light green), with SARS-CoV-2, or heat inactive (H.I) SARS-CoV-2. Full images of plaque forming unit assay using the viral permissive cell line, VeroE6-TMPRSS2 cells. Virus and heat inactive virus stocks were used as positive and negative controls (blue circles). Sketches of wells were made using BioRender.

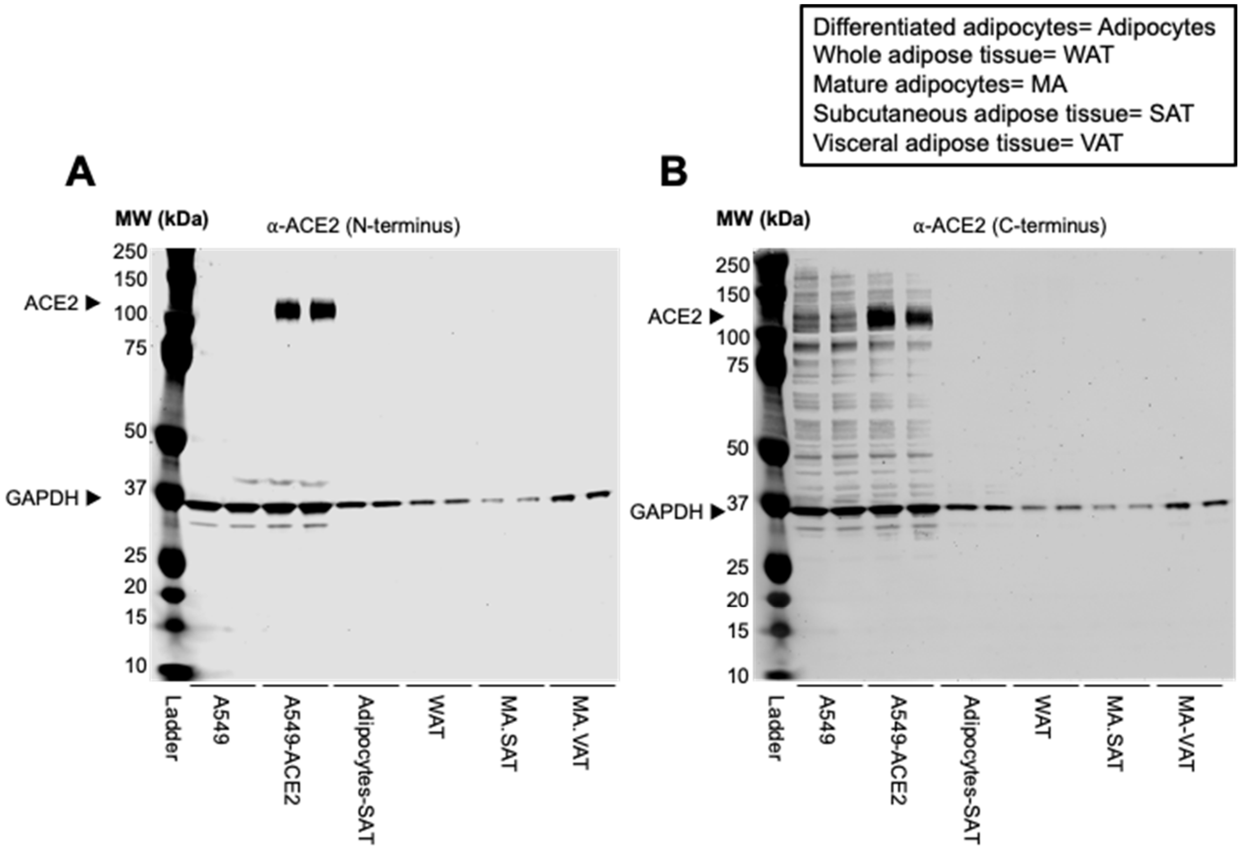


**Fig. S4. No ACE2 protein detection in adipose tissue cells.** Protein cell lysates were collected from in vitro differentiated adipocytes obtained from subcutaneous adipose tissue (Adipocytes-SAT) , fixed whole adipose tissue (WAT), and mature adipocytes collected from either subcutaneous (MA-SAT) or visceral (MA-VAT) adipose tissue. A549 and A549-ACE2 cell lysates were used as a negative and positive control, respectively, for ACE2 detection. Lysates were denatured and used for western blot analysis. (**A and B**) Protein-blocked membranes were stained with ACE2 antibodies targeting regions close to either the N (A) or C (B) terminus of human ACE2. GAPDH was used as a loading control. Fluorescence secondary antibodies were used to target the Fc portions of ACE2 (130 kDa) and GAPDH (37 kDa). Blots were read using the LI-COR Odyssey Near-IR scanner.

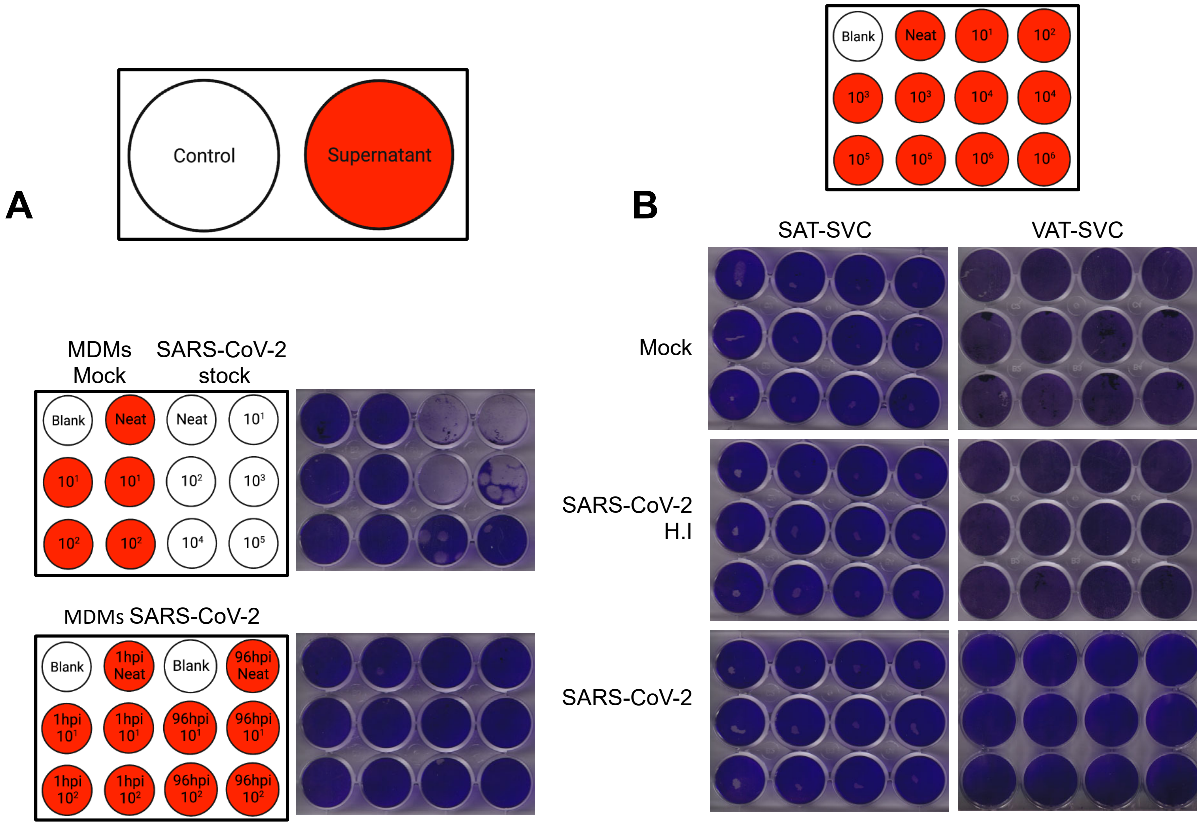


**Fig. S5. No plaque forming units were observed in cultures using supernatants from SVC or monocyte-derived macrophages exposed to SARS-CoV-2.** Stromal vascular cells (SVC) were obtained from fresh subcutaneous (SAT) or visceral (VAT) adipose tissue post-collagenase digestion. Monocyte-derived macrophages (MDM) were differentiated from blood monocytes for 7 days with macrophage colony-stimulating factor. Cells were infected, or mock infected, with SARS-CoV-2, or heat inactive (H.I) SARS-CoV-2. (**A and B**) Full images of plaque forming unit assay on 12-well plates using the viral permissive cell line, VeroE6-TMPRSS2. White circles represent control wells where virus stock or culture media (blank) was used. Red circles represent wells that were treated with supernatant from MDMs (A), SAT (B, left), or VAT (B, right). Sketches of wells were made using BioRender.

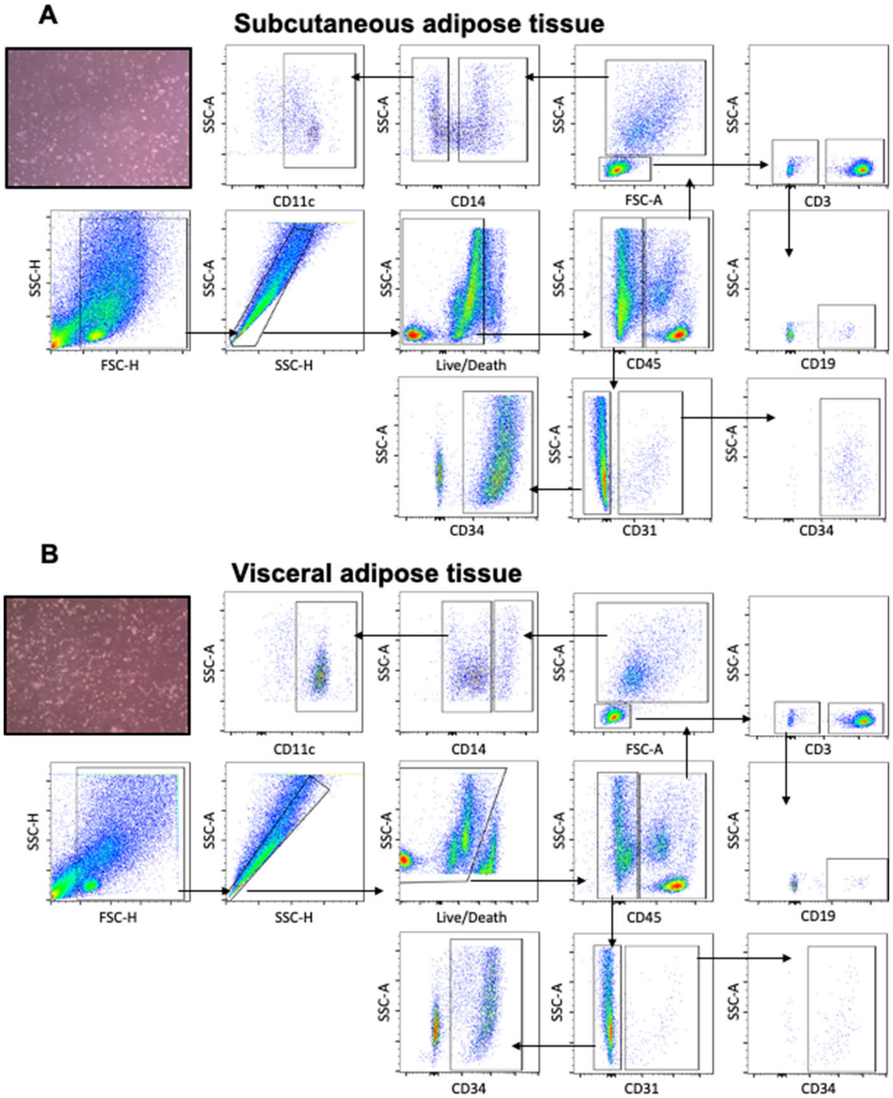


**Fig. S6. Gating strategy of SVC in human adipose tissue. (A and B)** Gating of SAT (A) and VAT (B) SVC started by debris removal using forward (FSC) and side scatters (SSC) followed by removal of doublets by SSC-area (A) and SSC-height (H) and selection of viable cells by negative population of zombie aqua dye. Cells selection was done as followed: Dendritic Cells ($CD45^+$→Myeloid→$CD14^-$→$CD11c^+$), Macrophages ($CD45^+$→Myeloid→$CD14^+$), B cells ($CD45^+$→lymphocyte→ $CD3^-$ → $CD19^+$), T cells ($CD45^+$→lymphocyte→ $CD3^+$), Endothelial Cells ($CD45^-$ → $CD31^+$→ $CD34^+$), and Preadipocytes ($CD45^-$ → $CD31^-$→ $CD34^+$). Flow cytometry data was collected using a 3-laser CYTEK Aurora; FCS files were analyzed in FlowJo10. Cell culture images were obtained with a EVOS XL Core Cell Imaging System on healthy SVC.

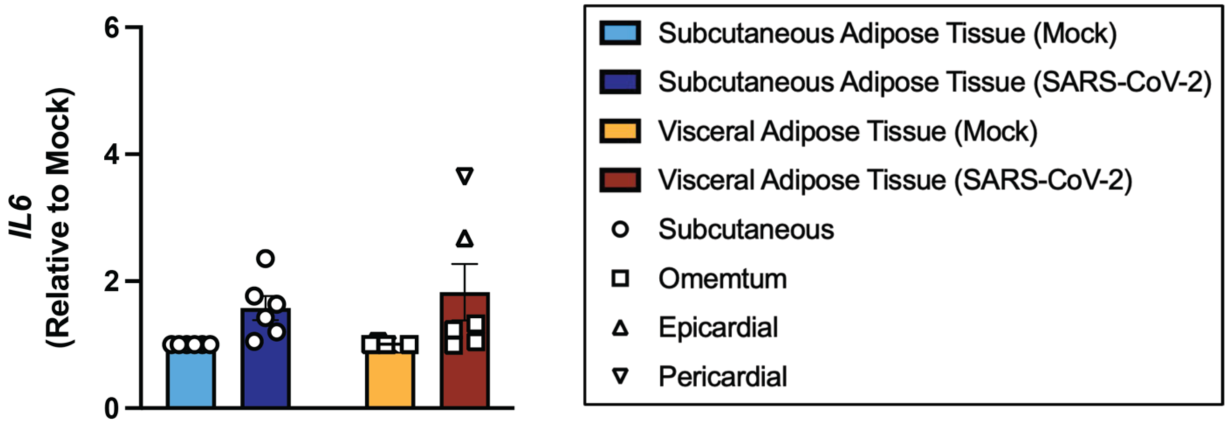


**Fig. S7. Elevated *IL6* gene expression in SVC post in vitro SARS-CoV-2 infection.** Stromal vascular cells (SVC) of human white adipose tissue were isolated by collagenase digestion prior to viral infection. SVC was infected or left untreated (mock) for 24 hours with SARS-COV-2 (USA-WA1/2020) at a MOI of 1. Relative gene expression of *IL6* (SAT, n=6; VAT, n=6) obtained by RTqPCR using eukaryotic *18S* rRNA as a housekeeping gene and analyzed by the ΔΔCt method relative to mock samples.

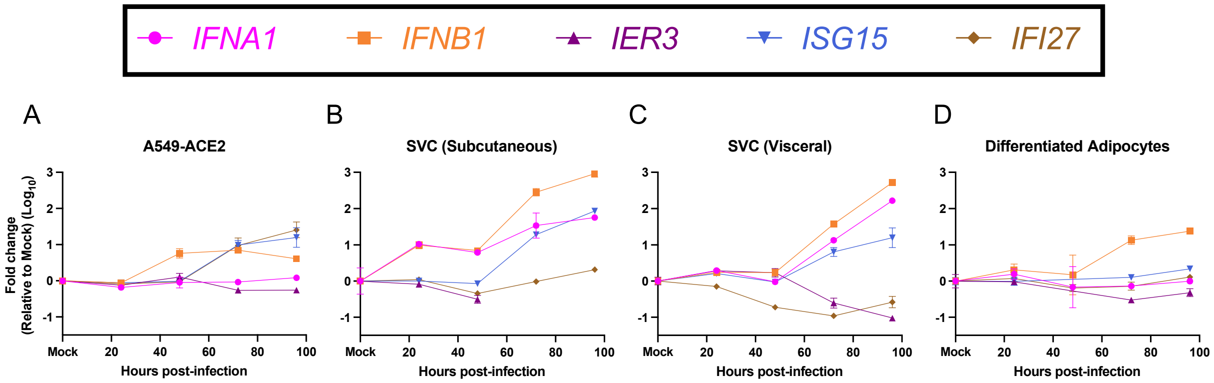


**Fig. S8. Increased interferon related genes in adipose tissue post-SARS-CoV-2 infection.** (**A**) A549-ACE2, (**B**) SAT-SVC, (**C**) VAT-SVC and (**D**) in vitro differentiated adipocytes (day 12, pericardium) were exposed to SARS-CoV-2 (MOI of 1) for 1 hour before washing and culturing for 24, 48, 72, and 96 hours. RNA was isolated and relative gene expression of *IFNA1, IFNB1, ISG15, IFI27, IER3* was obtained by 1-step RTqPCR using Taqman reagents. In all cases, *18s* was used as a housekeeping gene. Data is shown as fold change (log10) relative to mock sample. Undetected values were labeled as "no signal".

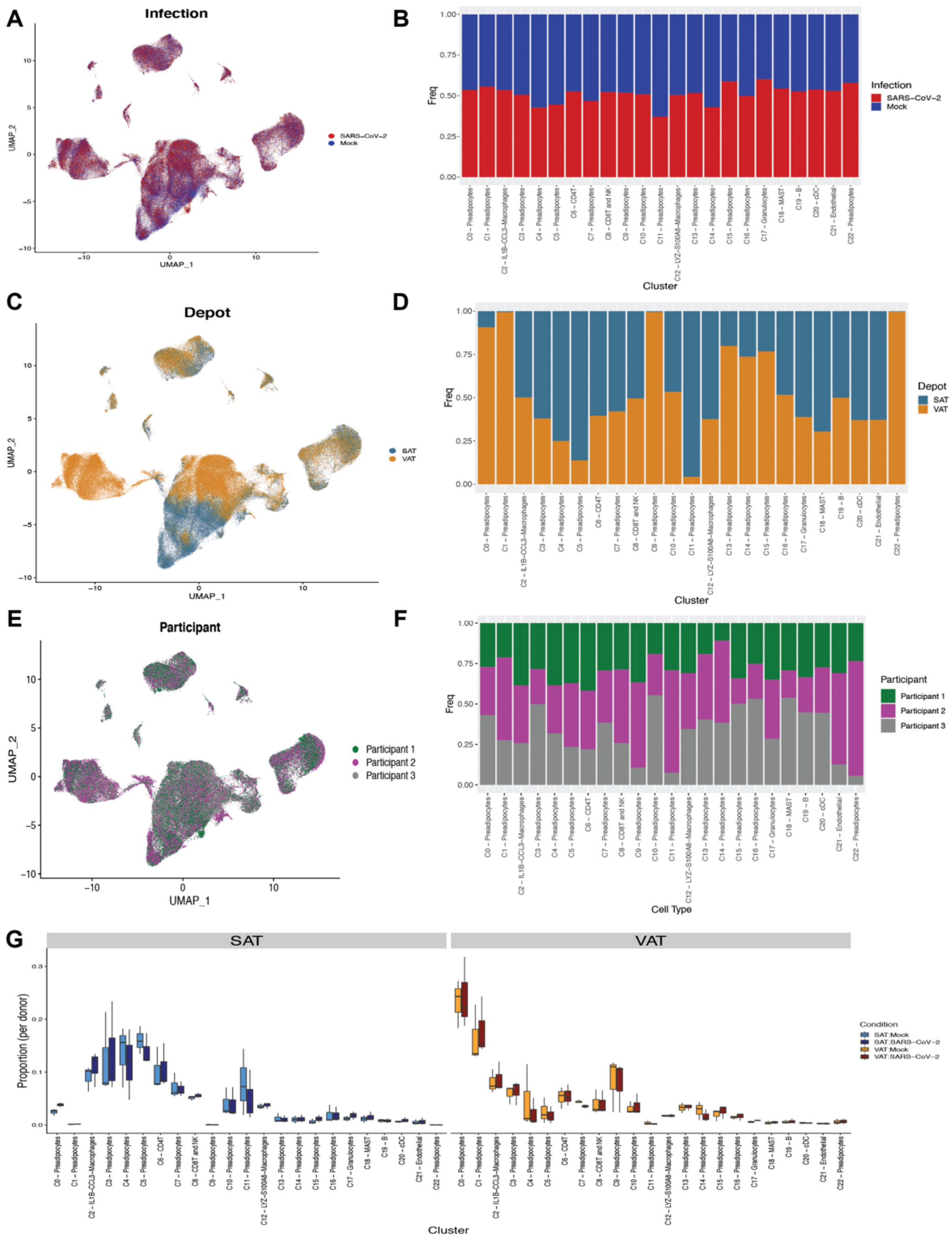


**Fig. S9. Infection, depot and participant breakdown by cluster annotation.** (**A to F**) UMAP representation of SVC (A, C, and E) and cell fraction barplot by cluster (B, D and F) from all participants (n=3) across 198,759 cells. Plots are colored by infection status (A and B), depot (C and D), and participant (E and F). (**G**) Barplot of the proportion of each cell type cluster within each participant by depot and infection status.

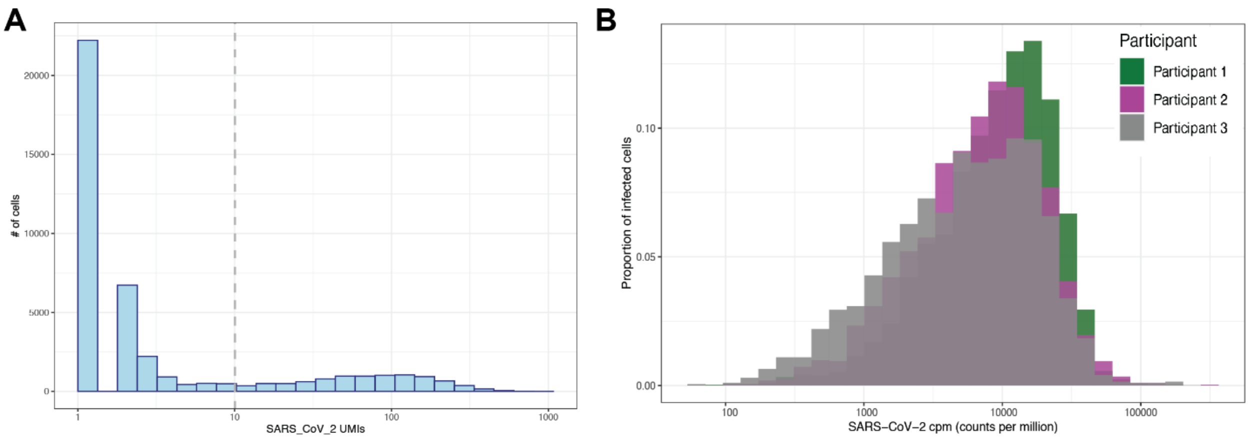


**Fig. S10. Distribution of SARS-CoV-2 transcripts across all cells.** (**A**) Distribution of SARS-CoV-2 UMIs across all cells with SARS-CoV-2 reads. The gray dotted line marks the 10 UMI threshold used throughout. (**B**) Distribution of all cells with SARS-CoV-2 reads above threshold, colored by participant and plotted by SARS-CoV-2 cpm (counts per million).

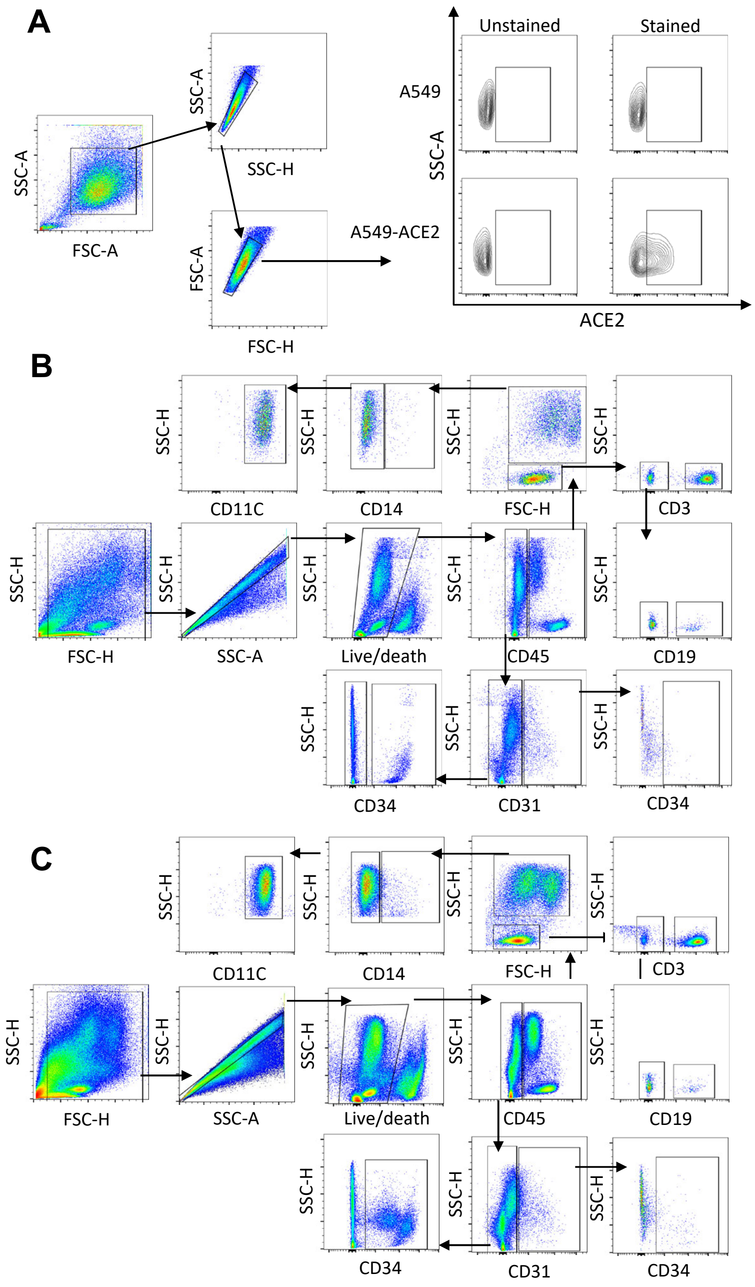


**Fig. S11. Limited ACE2 protein detection in SVC from SAT and VAT.** (**A**) Validation of human ACE2 antibody by comparing cell lines deficient in ACE2 expression (A549) to genetically modified ACE2-expressing A549 cells, A549-ACE2. (**A**) To the left, gating strategy and to the right panel comparing unstained to stained A549 and A549-ACE2 cells. (**B and C**) Gating strategy used to detect ACE2 protein in SVC from (B) SAT and (C) VAT.

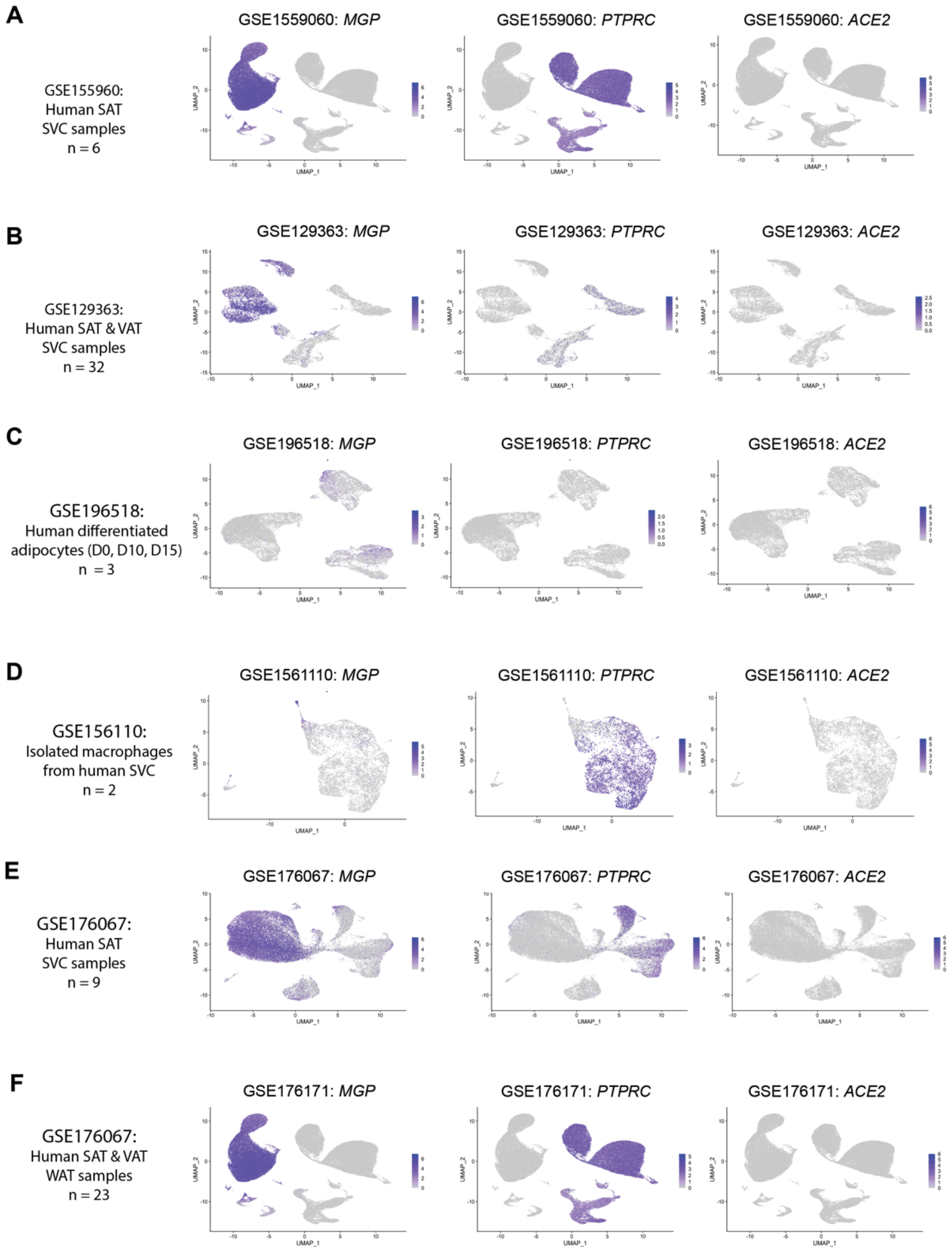


**Fig. S12.** ***ACE2*** **expression across public human adipose single-cell RNA-sequencing datasets.** (**A to F**) UMAP projections of all cells from four different public human adipose scRNA seq dataset, colored by *MGP*, *PTPRC* and *ACE2* expression. The datasets analyzed include (A) GSE155960, (B) GSE129363, (C) GSE196518 (D) GSE156110, (E) GSE196518 and (F) GSE156610 (single-nucleus sequencing).

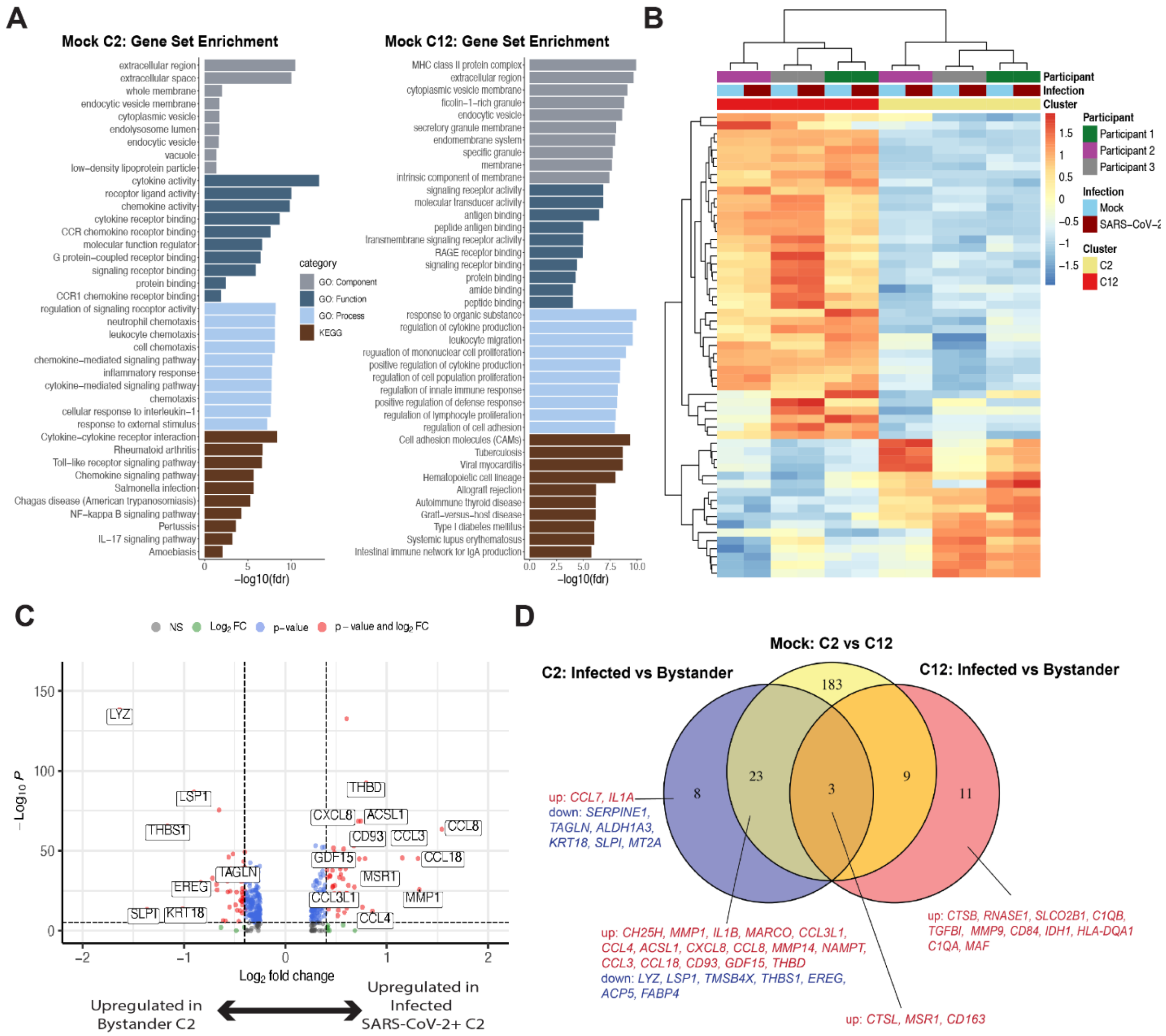


**Fig. S13. C2- and C12-macrophages are distinctly different upon mock and SARS-CoV-2 infection.** (**A**) Heatmap of the significant differentially expressed genes (DEGs; padj < .05, abs(log2FC) >= 0.6) between mock C2- and C12-macrophages colored by z-score across all participants and infection conditions. (**B**) Gene ontology (GO)-term and Kyoto Encyclopedia of Genes and Genomes (KEGG) pathway enrichment of the up-regulated genes derived from fig. S8A in C2 macrophages (74 DEGs) and C12 macrophages (146 DEGs). Top 10 pathways per pathway category with a false discovery rate (FDR) < 0.05 across each pathway category were included and the -log (FDR) was plotted per pathway. (**C**) Volcano plot of the DEGs between infected (SARS-CoV-2+) versus bystander C2-macrophages in SARS-CoV-2 infected SAT and VAT. ns, not significant. (**D**) Venn diagram comparing the significant DEGs (padj < .01, abs(log2FC) > 0.6) with direction of change taken into account, across SARS-CoV-2+ infected macrophages versus bystander macrophages within the C2-macrophage population and within the C12-macrophage population of SARS-CoV-2 infected samples. DEGs between C2 and C12 across mock-infected samples were also included for comparison.

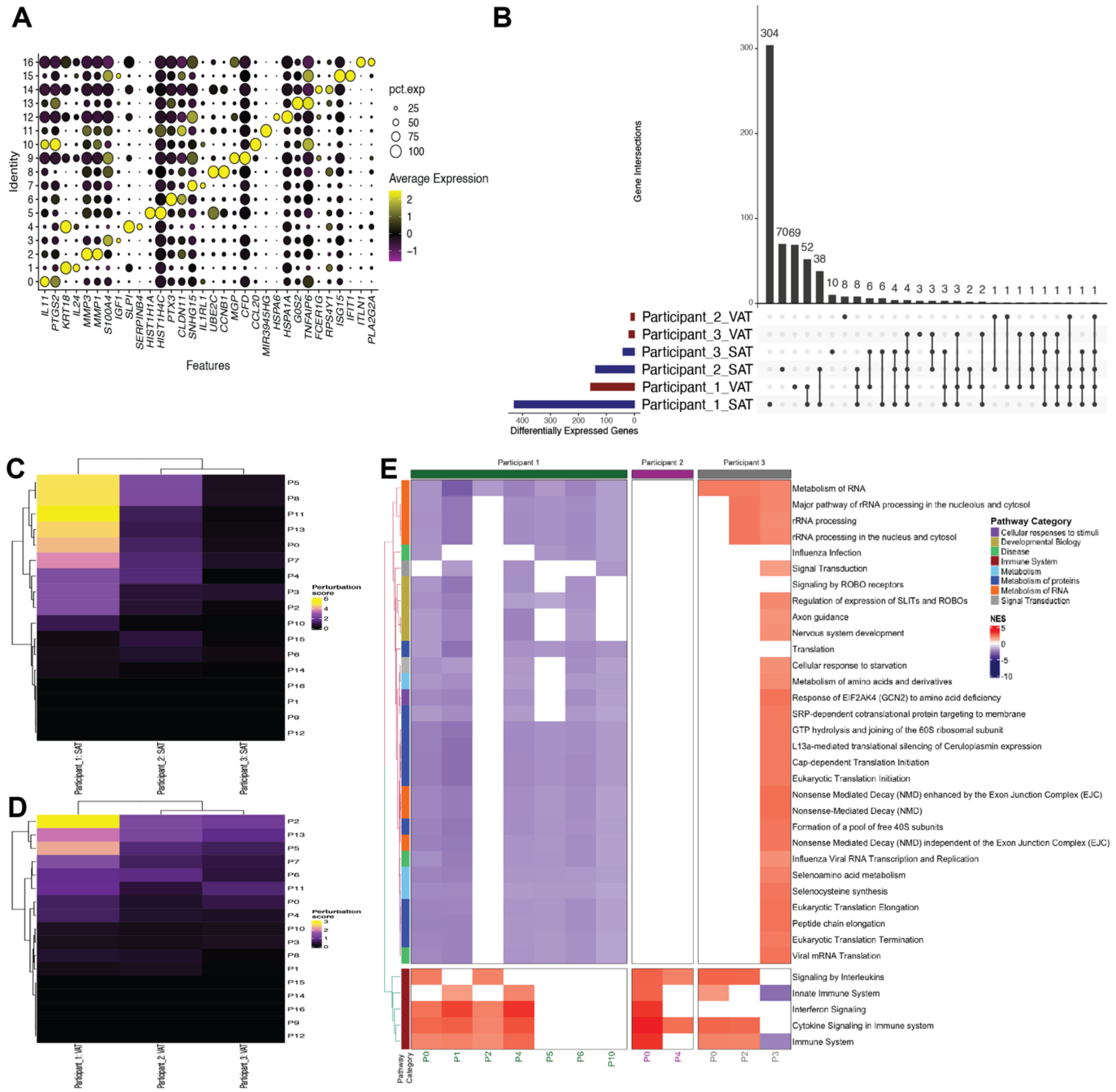


**Fig. S14. Characterization of preadipocytes across VAT and SAT.** (**A**) Dotplot of the top 2 cluster defining genes per preadipocyte cluster colored by scaled average expression and size representing the percent of cells in the cluster expressing the given transcript (pct.exp). (**B**) Upset plot showing the DEGs in the preadipocytes of SARS-CoV-2 infected samples versus mock-infected samples. Left bar graph depicts the number of differentially expressed genes per depot and participant between the SARS-CoV-2 infected sample versus the mock-infected sample. The matrix depicts whether the DEGs are unique to the participant-depot or shared across multiple participant-depots. Bar graph at top demonstrates the number of DEGs that are associated with each participant-depot combination denoted by the matrix. (**C and D**) Perturbation analysis was performed by

taking each preadipocyte cluster and first identifying the DEGs between SARS-CoV-2 infected samples versus the mock-infected samples and then projecting the whole transcriptome of each participant onto this gene set. Heatmap of the perturbation scores per participant are displayed for both the (C) SAT and (D) VAT depots. (**E**) Reactome pathway analysis was performed on the significant DEGs (padj < 0.1) between SARS-CoV-2-exposed versus mock preadipocytes by participant and cluster within VAT. Pathways that were represented and significant (padj < 0.05) in at least four of the participant-cluster subsets were included. Pathways clustered by euclidean distance (tree not shown) and split by the two major subtrees. NES, normalized enrichment score.

**Table S1. Adipose tissue participant's demographic, medical information, and sample use.** Table summarizing all adipose tissue participant information from samples used in this study. The information presented is the following: (left to right) participant ID, age, sex, ethnicity, if patient had type 2 diabetes (T2DM) or hypertension (HT), body mass index (BMI), other risk factors, type of adipose tissue obtained, and experiments performed on adipose tissue.

**Table S1: Adipose tissue donor's demographic, medical information, and sample use**

| ID | Age | Sex | Ethnicity | T2DM/HTN | BMI | Medications | Other risk factors | Adipose depot | Experiment performed |
|---|---|---|---|---|---|---|---|---|---|
| 531 | 59 | M | W | Y/Y | 41 | Metformin, Lantus, Ozempic, Jardiance, Atenolol, and Lisinopril | | SAT and VAT | RTqPCR (*ACE2*, SARS-CoV-2), RTqPCR (ACE2) on differentiated adipocytes |
| 888 | 49 | F | W | Y/Y | 52 | Dulaglutide, Empagliflozin, Metformin, Carvedilol, Anastrozole, and Lisinopril | CHF | SAT and VAT | Used to develop experimental protocols |
| 108 | 36 | F | H | N/Y | 47 | Amlodipine, Lisinopril, and Hydrochlorothiazide | Asthma | SAT and VAT | Used to develop experimental protocols |
| 935 | 58 | F | W | N/N | 37 | None | | SAT and VAT | RTqPCR (*ACE2*) on differentiated adipocytes |
| 159 | 52 | M | H | N/N | 40 | Liraglutide and Metformin | | SAT and VAT | Used to test antibodies for Flow cytometry panel |
| 120 | 33 | F | H | N/N | 52 | None | | SAT and VAT | Luminex, RTqPCR (*ACE2*, *IL6*, SARS-CoV-2) |
| 611 | 52 | F | W | N/Y | 46 | Valsartan and Hydrochlorothiazide | | SAT and VAT | Luminex, Flow cytometry (SARS-CoV-2) |
| 959 | 54 | F | W | N/Y | 40 | Valsartan and Hydrochlorothiazide | | SAT and VAT | Luminex |

| | | | | | | | | | |
|---|---|---|---|---|---|---|---|---|---|
| 608 | 42 | M | W | N/Y | 52 | Metformin and Liraglutide | | SAT and VAT | Luminex, Flow cytometry (SARS-CoV-2) |
| 002 | 67 | F | W | Y/Y | 30.1 | Glyburide, Metformin, Hydrochlorothiazide, and Lopressor | Asthma and CAD | EAT, PAT, and SAT | Used to develop experimental protocols |
| 003 | 61 | M | H | N/Y | 30.8 | Labetalol, Valsartan, and Amlodipine | CHF and CAD | EAT, PAT, and SAT | RTqPCR (SARS-CoV-2) on differentiated adipocytes |
| 004 | 72 | F | H | Y/Y | 33.2 | Amlodipine and Lopressor | ESRD on HD and CAD | EAT, PAT, and SAT | RTqPCR (*ACE2*) on differentiated adipocytes |
| 005 | 63 | M | H | Y/Y | 29.4 | Canagliflozin, Glipizide, Insulin glargine, Metformin, Benazepril, Furosemide, and Lopressor | Smoker and CAD | EAT, PAT, and SAT | Luminex, RTqPCR (*ACE2*, *IL6*, SARS-CoV-2) |
| 319 | 61 | F | W | N/N | 32.8 | None | | SAT | Luminex |
| 209 | 35 | F | H | N/N | 38.4 | None | | SAT and VAT | Flow cytometry (ACE2) |
| 272 | 36 | F | A | N/Y | 42.8 | Losartan | | SAT and VAT | Luminex |
| 658 | 60 | F | H | Y/N | 38.1 | Metformin and Azathioprine | COPD, Asthma, Obesity, Smoker, and MG | SAT and VAT | scRNA-seq (Participant 1), Luminex, and RTqPCR (SARS-CoV-2, and *IL6*) |
| 576 | 33 | M | H | Y/Y | 47.9 | Metformin | | SAT and VAT | scRNA-seq (Participant 2), Luminex, and RTqPCR (SARS-CoV-2, and *IL6*) |

| | | | | | | | | | |
|---|---|---|---|---|---|---|---|---|---|
| 924 | 50 | F | B | N/Y | 54.5 | Insulin glargine, Insulin lispro, Losartan, Furosemide, and Carvedilol | T1DM, severe obesity, former smoker | SAT and VAT | scRNA-seq (Participant 3), Luminex, and RTqPCR (SARS-CoV-2, and IL-6) |
| 854 | 34 | F | H | N/N | 41.6 | None | Asthma | SAT and VAT | Plaque assay and RTqPCR (SARS-CoV-2) |
| 754 | 23 | F | B | N/N | 39.2 | None | Asthma | SAT and VAT | RTqPCR (SARS-CoV-2), Inoculation of cell line for cytopathic effect |

**Abbreviations:** type 1 diabetes mellitus (T1DM), male (M), female (F), yes (Y), no (N), white (W), black (B), Hispanic (H), Asian (A), congestive heart failure (CHF), coronary artery disease (CAD), end-stage renal disease (ESRD), hemodialysis (HD), and chronic obstructive pulmonary disease (COPD), myasthenia gravis (MG).

**Table S2. Antibodies and reagents used in flow cytometry**

| Antigen | Isotype/Host | Fluorochrome | Clone | Company | Catalog number | Antibody dilution |
|---|---|---|---|---|---|---|
| CD45 | IgG1,k/Mouse | PERCP-CY 5.5 | 2D1 | BioLegend | 368503 | 1:100 |
| CD3 | IgG2a,k/Mouse | BV421 | OKT3 | BioLegend | 317343 | 1:100 |
| CD14 | IgG2a,k/Mouse | BV650 | M5E2 | BioLegend | 301835 | 1:100 |
| CD19 | IgG1,k/Mouse | BV750 | HIB19 | BioLegend | 302261 | 1:100 |
| CD11c | IgG2b,k/Mouse | PE | S-HCL-3 | BioLegend | 371503 | 1:100 |
| CD34 | IgG2a,k/Mouse | PE-CY7 | 561 | BioLegend | 343615 | 1:100 |
| CD31 | IgG1,k/Mouse | AF700 | WM59 | BioLegend | 303133 | 1:100 |
| ACE2 | IgG2a,k/Mouse | AF647 | 535919 | R&D | fab9332r | 1:50 |
| Isotype control | IgG2a,k/Mouse | AF647 | 20102 | R&D | ICOO3R | 1:100 |
| SARS-CoV-2 (N protein)* | IgG/Rabbit | AF647 | N/A | Sino Biological | 40143-T62 | 1:50 |

**Note:** * antibody was conjugated in-house.

**Supplementary Data Files**

Data file S1. 80-plex Luminex data.

Data file S2. Marker genes for each cluster defined in combined Seurat analysis.

Data file S3. Differentially expressed transcripts within macrophages only, across different subsets of infection, mock and depot.

Data file S4. GO and KEGG term enrichment for markers of C12 macrophages.

Data file S5. GO and KEGG term enrichment for markers of C2 macrophages.

Data file S6. Differentially expressed transcripts between infected (SARS-CoV-2+) versus bystander populations within the C2 and C12 infected macrophage populations.

Data file S7. Reactome pathways for differentially expressed genes between infected (SARS-CoV-2+) versus bystander C2 macrophages.

Data file S8. Marker genes for each cluster defined from the Seurat analysis of Preadipocytes only.

Data file S9. ISG and cytokine gene modules.

Data file S10. Differentially expressed genes between SARS-CoV-2 exposed versus mock exposed preadipocytes, by cluster and participant, within the SAT

Data file S11. Differentially expressed genes between SARS-CoV-2 exposed versus mock exposed preadipocytes, by cluster and participant, within the VAT.

Data file S12. Reactome pathways for differentially expressed genes between SARS-CoV-2 exposed versus mock exposed preadipocytes, by cluster and participant, within the SAT.

Data file S13. Reactome pathways for differentially expressed genes between SARS-CoV-2 exposed versus mock exposed preadipocytes, by cluster and participant, within the VAT.

Data file S14: Figure raw data.
